# Overexpression of kinesin superfamily members as prognostic biomarkers of breast cancer

**DOI:** 10.1186/s12935-020-01191-1

**Published:** 2020-04-15

**Authors:** Tian-Fu Li, Hui-Juan Zeng, Zhen Shan, Run-Yi Ye, Tuck-Yun Cheang, Yun-Jian Zhang, Si-Hong Lu, Qi Zhang, Nan Shao, Ying Lin

**Affiliations:** 1 Breast Disease Center, The First Affiliated Hospital of Sun Yat-sen University, Guangzhou, 510080 China; 2 Laboratory of Surgery, The First Affiliated Hospital, Sun Yat-Sen University, Guangzhou, 510080 China; 3 Guangdong Key Engineering Laboratory for Diagnosis and Treatment of Vascular Disease, The First Affiliated Hospital, Sun Yat-Sen University, Guangzhou, 510080 China

**Keywords:** Kinesin superfamily, Breast cancer, Prognostic biomarker, MSX1, Bioinformatics analysis

## Abstract

**Background:**

Kinesin superfamily (KIFs) has a long-reported significant influence on the initiation, development, and progress of breast cancer. However, the prognostic value of whole family members was poorly done. Our study intends to demonstrate the value of kinesin superfamily members as prognostic biomarkers as well as a therapeutic target of breast cancer.

**Methods:**

Comprehensive bioinformatics analyses were done using data from TCGA, GEO, METABRIC, and GTEx. LASSO regression was done to select tumor-related members. Nomogram was constructed to predict the overall survival (OS) of breast cancer patients. Expression profiles were testified by quantitative RT-PCR and immunohistochemistry. Transcription factor, GO and KEGG enrichments were done to explore regulatory mechanism and functions.

**Results:**

A total of 20 differentially expressed KIFs were identified between breast cancer and normal tissue with 4 (KIF17, KIF26A, KIF7, KIFC3) downregulated and 16 (KIF10, KIF11, KIF14, KIF15, KIF18A, KIF18B, KIF20A, KIF20B, KIF22, KIF23, KIF24, KIF26B, KIF2C, KIF3B, KIF4A, KIFC1) overexpressed. Among which, 11 overexpressed KIFs (KIF10, KIF11, KIF14, KIF15, KIF18A, KIF18B, KIF20A, KIF23, KIF2C, KIF4A, KIFC1) significantly correlated with worse OS, relapse-free survival (RFS) and distant metastasis-free survival (DMFS) of breast cancer. A 6-KIFs-based risk score (KIF10, KIF15, KIF18A, KIF18B, KIF20A, KIF4A) was generated by LASSO regression with a nomogram validated an accurate predictive efficacy. Both mRNA and protein expression of KIFs are experimentally demonstrated upregulated in breast cancer patients. Msh Homeobox 1 (MSX1) was identified as transcription factors of KIFs in breast cancer. GO and KEGG enrichments revealed functions and pathways affected in breast cancer.

**Conclusion:**

Overexpression of tumor-related KIFs correlate with worse outcomes of breast cancer patients and can work as potential prognostic biomarkers.

## Introduction

Worldwide, breast cancer raises concerns to human health, women especially, with continuously increasing incidence and high mortality. 2.1 million new cases diagnosed and 626,679 deaths found in 2018 make breast cancer the most commonly diagnosed cancer and the leading cause of cancer death in women [1]. Great efforts are put by clinicians and researchers and progressions are seen in early detection, diagnosis, and treatments of breast cancer over the years with a significant extension of breast cancer survival [2]. Nevertheless, early recurrence, distant metastasis and drug resistance are still commonly seen, which hold threads to the prognosis of breast cancer patients and mount challenges for clinicians [3–5]. Further researches were urgently needed to unravel the molecular mechanism underlying and discovering valuable prognostic biomarkers for breast cancer survival.

Kinesin superfamily (KIFs) were a group of proteins featured to be microtubule-based motors and functioned as intracellular transporters that directionally transport various cargos, including organelles, protein complexes and mRNAs, along microtubules in an adenosine triphosphate (ATP)-dependent way and played crucial roles in not only cellular morphogenesis and fundamental biology, like mitosis and meiosis, but also various mechanisms for higher life functions, including higher brain functions like memory and learning, left–right asymmetry formation, etc. [6–8]. There are 45 KIFs discovered and identified in human, among which several family members were demonstrated varied functions in tumor pathobiology [9]. KIF11 was identified as a molecular target that shuttles between the proliferation and invasion of glioblastoma. Administration of KIF11 inhibitors in glioblastoma-bearing mice had a significantly extended survival indicating a putative therapeutic target for glioblastoma [10]. KIF20A peptide-based immunotherapy for cancer treatment was demonstrated availability and putative efficacy with promiscuous T_-H_-cell epitopes derived from KIF20A identified in solid tumor tissue and distinguished KIF20A-specific T_H_1-cell responses were found in patients with HNMT receiving immunotherapy [11]. Microarray data analyses revealed the highly transactivated status of KIF4A in non-small cell lung cancer and targeting KIF4A might hold a promise for the development of anticancer drugs and cancer vaccines as well as a prognostic biomarker in the clinic [12]. Numerous researches were done highlighting the importance of KIFs in various aspects of breast cancer [13]. KIF2A, KIF14 and KIF26B were found overexpressed in lymph nodes-positive breast cancer patients indicating putative impacts on tumor metastasis [14–16]. Knocking down of KIF2C, KIF3C, KIF22, KIF18A and KIF24 inhibited proliferation of breast cancer cells via different mechanisms including G2/M phase arrest, delayed exit from mitosis, deregulating cell division and restoring ciliation [17–22]. Recent researches demonstrated implications of KIF1A, KIF5A, KIF12, KIF14, KIFC1 and KIFC3 in resistance to docetaxel by destabilizing microtubule [23–26], while KIF5A, KIF5B, KIF12, KIF20A and KIFC3 were found to reduce the efficacy of paclitaxel by inducing abnormal breakdown of microtubules in breast cancer treatment [24, 27–29].

Given the essential roles of KIFs reported in cancer, KIF-targeting cancer therapies were highly expected to be of great efficacy and several KIF-inhibitors were invented and tested in clinical trials. Ispinesib, a KIF11-targeted inhibitor, was the first KIF-inhibitor that was evaluated both safety and efficacy in breast cancer in phase I clinical study [30]. Other KIF-targeted drugs further tested in various cancers by clinical trials including KIF11 inhibitors (litronesib [31, 32], filanesib [33–35], SB-743921 [36], AZD4877 [37]), KIF5C inhibitors (Lidocaine and Tetracaine [38]) and KIFC1 inhibitors (AZ82 and SR31527 [39, 40]). However, limited efficacy was seen in all inhibitors reported. Therefore, despite numerous researches done, the prognostic and therapeutic value of all KIFs remains uncorroborated. Considering the intricate functions of KIFs in mitosis, singling out any particular KIFs may not be an efficient way to fulfill the therapeutic capacity of KIFs, while common regulatory network of all KIFs are little known, which may give new insight into the limited therapeutic efficacy shown in clinical trials and provide putative drug target by mutually regulating KIFs in cancer.

By adopting comprehensive multi-dataset bioinformatics analyses, our study intends to demonstrate the value of kinesin superfamily members as prognostic biomarkers of breast cancer, explore the putative regulatory network of KIFs, discover common functions and pathways shared among members and provide promising insights into breast cancer treatment.

## Methods and materials

### Patient samples

All clinical samples were collected from the first affiliated hospital of Sun Yat-sen University from March to April 2019. Inclusion criteria were primary breast cancers with solid pathological diagnosis from one pathologist and proceeded whole-journey diagnosis, operation and post-operational treatment. Patients with distance metastasis at first diagnosis or earlier treatment procedures were excluded. The cancer nuclear grade was done according to the Nottingham Histologic Score system and stage was done according to the AJCC 8th anatomic stage system. Breast cancer tissues and paired paratumor tissues were all taken from fresh operation samples and separated within 30 min after removal. Liquid nitrogen was used for immediate restoration and subsequently long-term cryopreservation was done at − 80 °C in the refrigerator until RNA extraction. Samples used in this study were approved by the Committees for Ethical Review of Research involving human subjects at the First Affiliated Hospital, Sun Yat-Sen University.

### Expression analysis

KIFs RNA sequencing expression data of breast cancer from the cancer genome atlas (TCGA, http://can-cergenome.nih.gov/) were downloaded using UCSC XENA data hubs (https://tcga.xenahubs.net), the heatmap was drawn using R package “pheatmap” with clinical parameters from TCGA-BRCA dataset [41]. Expression comparisons of 810 breast tumors and 291 normal samples from TCGA and the GTEx projects were done using GEPIA 2 (http://gepia2.cancer-pku.cn/) standard processing pipeline [42].

### Survival analysis

Relapse-free survival (RFS), overall survival (OS), distant metastasis-free survival (DMFS) for all KIFs were done using KM plotterr with endpoint definition previously reported (http://kmplot.com/) [43]. Both RNA-seq data from TCGA and chip-seq data from gene expression omnibus (GEO, https://www.ncbi.nlm.nih.gov/ geo/) and Molecular Taxonomy of Breast Cancer International Consortium (METABRIC) project were used for Kaplan–Meier-plot [44]. Specific chip-seq datasets used include E-MTAB-365, GSE11121, GSE12093, GSE12276, GSE1456, GSE16391, GSE16446, GSE16716, GSE17705, GSE17907, GSE19615, GSE20271, GSE2034, GSE20685, GSE20711, GSE21653, GSE2603, GSE26971, GSE2990, GSE31519, GSE3494, GSE37946, GSE42568, GSE45255, GSE4611, GSE4922, GSE5327, GSE6532, GSE7390, GSE9195. Auto-selected best cutoffs were adopted for estimation of prognostic value.

### LASSO regression

LASSO Cox regression is a widely-used method for high-dimensional predictors selection [45]. In this study, TCGA-BRCA data were used to construct a prognostic model of KIFs for the prediction of OS. R package “glmnet” was used to execute LASSO Cox regression model analysis [46]. Cvfit plot was drawn and the minimum lambda value was used as cutoff. The predictive model was validated in TCGA-BRCA and chip-seq data using KM plotter as previously described.

### Multivariate survival analysis

Multivariate survival analysis of RFS, OS and DMFS were done using chip-seq data to discover KIFs related clinical and molecular-pathological characteristics. Elements evaluated including ER, PR, HER-2 status, lymph node status, tumor grade, intrinsic subtype, TP53 status, endocrine therapy and chemotherapy history.

### Nomogram

A nomogram predicting 5-year and 8-year overall survival of breast cancer patients was constructed combining clinical, molecular-pathological characteristics and LASSO regression generated KIFs model using R package “rms” [47, 48]. Assessment of predictive accuracy was done by calibration plot with self-validation done every 80 patients itinerantly for better stability [49].

### Total RNA extraction and qRT-PCR

RNA was isolated from tumor and paratumor tissues using an RNA extraction kit (Promega, Beijing, China). Single-stranded cDNA was generated from 1ug total RNA in a 20 μl reaction volume with 4 μl RT reagent (Takara, Japan). The quantitative real-time PCR reaction was performed with the SYBR green detection (Penzberg, German). GAPDH was used as an endogenous control. The relative expression levels were measured by qRT-PCR using LightCycle 480 II (Roche, Switzerland). Each of the experiments was performed in triplicate. The primer pairs for each target gene were listed in Additional file 1.

### Immunohistochemistry

Immunohistochemistry images of KIFs in normal breast tissue from the GTEx project and tumor tissue from TCGA-BRCA were obtained from the human protein atlas (https://www.proteinatlas.org) [50–54]. All IHC staining in the Human Protein Atlas project is performed using a standard protocol as described previously [52, 54]. Annotation parameters include an evaluation of (i) staining intensity (negative, weak, moderate or strong), (ii) fraction of stained cells (rare, < 25%, 25–75% or > 75%) and (iii) subcellular localization (nuclear and/or cytoplasmic/membranous).

### Enrichment of co-expression genes, transcription factors, GO and KEGG

Co-expression genes correlated with KIFs were enriched using R2: Genomics Analysis and Visualization Platform (http://r2.amc.nl) TCGA-BRCA data, separately. P-value cutoff < 0.001 and correlation R-value > 0.5 were set as cutoffs. Intersections of KIFs-related genes were calculated by the upset plot. The intersection of co-expressed genes was put into GO, KEGG and transcription factors enrichment using R package “ClusterProfiler” [55]. For GO and KEGG enrichment, P-value < 0.001 and Q-value < 0.01 were used as cutoffs and enrichments were done for all three GO categories. For transcription factors enrichment, P value < 0.05 and Q value < 0.05 were used as cutoff. Enrichment results were represented as bubble plot, chord plot and cluster heatmap plot using R package “GOplot” [56].

### Statistics

For all the analyses done above, a P-value < 0.05 was considered statistically significant except for specifically mentioned.

## Results

### Expression profile of KIFs in breast cancer

**Fig. 1:**
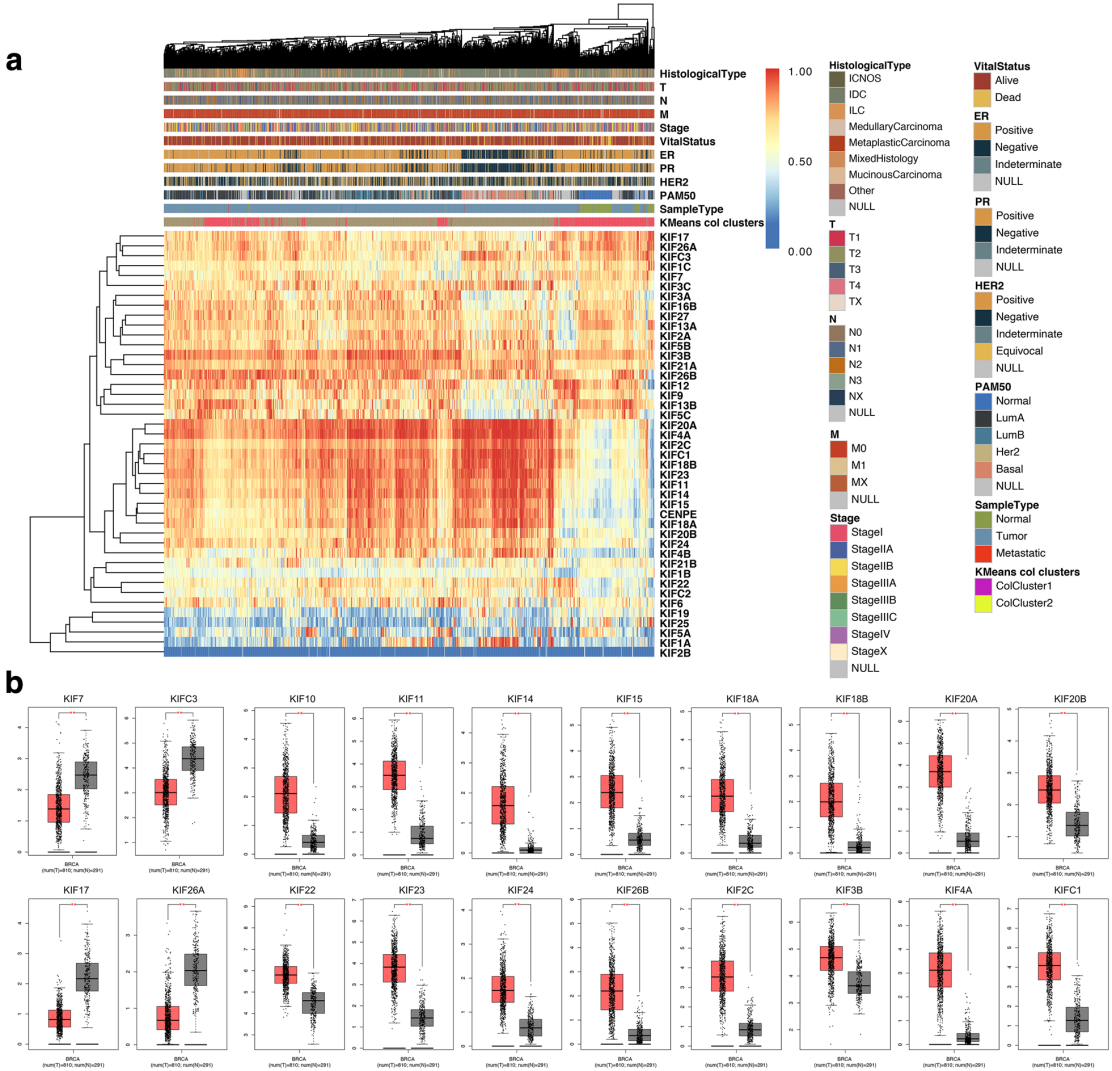
Expression profiles of kinesin superfamily in breast cancer. **a** Expression and clinical characteristics of all kinesin superfamily members using TCGA-BRCA data (Normal = 113, Metastatic = 7, Tumor = 1097). **b** Expression of each under-expressed kinesin superfamily members (KIF17, KIF26A, KIF7, KIFC3) and overexpressed kinesin superfamily members (KIF10, KIF11, KIF14, KIF15, KIF18A, KIF18B, KIF20A, KIF20B, KIF22, KIF23, KIF24, KIF26B, KIF2C, KIF3B, KIF4A, KIFC1) using both TCGA-BRCA and matched GTEx-breast normal data (Tumor = 810; Normal = 291); *P < 0.05; **P < 0.001

The TCGA expression profile of all KIFs was shown in the heatmap (Fig. 1a). Distinct expressions profiles were seen between normal and tumor tissue. Clustering analysis found most KIFs overexpressed in tumor tissue. Owing to the lack of normal samples, further comparisons were done between TCGA tumor samples and matched TCGA and GTEx normal samples. A total of 20 differentially expressed KIFs were found. Among which, only 4 KIFs (KIF17, KIF26A, KIF7, KIFC3) showed decreased expression in tumor samples while 16 KIFs (KIF10, KIF11, KIF14, KIF15, KIF18A, KIF18B, KIF20A, KIF20B, KIF22, KIF23, KIF24, KIF26B, KIF2C, KIF3B, KIF4A, KIFC1) significantly overexpressed in breast cancer (Fig. 1b, P < 0.001). Except for sample type, further explorations between clinical parameters and KIFs expression found stable expression patterns of the 20 differentially-expressed KIFs between tumor and normal samples in all four subtypes of breast cancer (Fig. 1a, Additional file 2). However, within tumor samples, identical expression patterns were seen in KIF10, KIF11, KIF14, KIF15, KIF18A, KIF18B, KIF20A, KIF20B, KIF23, KIF4A and KIFC1 with significantly high expression in Basal-like and Luminal B subtypes comparing to Luminal A breast cancer (Fig. 1a, Additional file 3).

### Overexpression of survival-related KIFs indicate worse outcomes in breast cancer

**Fig. 2:**
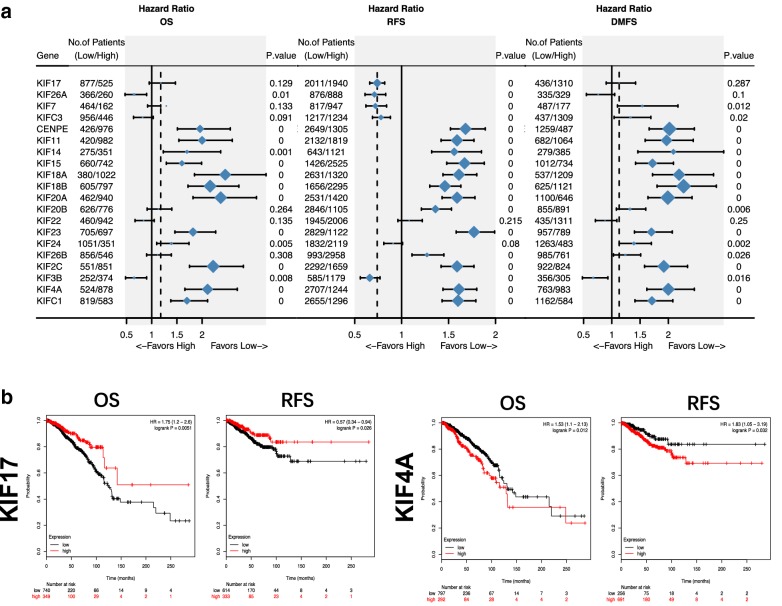
Survival analyses of 20 deregulated kinesin superfamily in breast cancer. **a** Forrest plot of 20 deregulated KIFs (KIF17, KIF26A, KIF7, KIFC3, KIF10, KIF11, KIF14, KIF15, KIF18A, KIF18B, KIF20A, KIF20B, KIF22, KIF23, KIF24, KIF26B, KIF2C, KIF3B, KIF4A, KIFC1) with survival analyses regarding OS, RFS and DMFS using chip-seq data. **b** Survival analyses of KIF17 and KIF4A regarding OS and RFS using TCGA-BRCA dataset. Red: high expression group; black: low expression group. P value was log-ranked. Auto-selected best cutoff were used

By combining chip-seq data from GEO and METABRIC, a large cohort with 3951 patients was used to explore the prognostic value of all KIFs in breast cancer using Kaplan–Meier plot. Significance were found in all 20 differentially-expressed KIFs regarding either OS, RFS or DMFS except for KIF22, while overexpression of 11 KIFs (KIF10, KIF11, KIF14, KIF15, KIF18A, KIF18B, KIF20A, KIF23, KIF2C, KIF4A, KIFC1) were found to be significantly related to worse outcomes regarding OS, RFS and DMFS, indicating that KIFs play important roles in breast cancer and hold the key to the prognosis of patients (Fig. 2a). Further explorations were done using TCGA data. Overexpression of KIF17 showed prognostic value of indicating better OS [HR = 1.75 (1.2–2.6), logrank P = 0.0051] and RFS [HR = 0.57 (0.34–0.94), logrank P = 0.026] while KIF4A demonstrated significant correlations with worse outcomes [OS: HR = 1.53 (1.1–2.13), logrank P = 0.012; RFS: HR = 1.83 (1.05–3.19), logrank P = 0.032] (Fig. 2b, Additional files 4, 5).

### Construction of 6-KIFs-based risk score

**Fig. 3:**
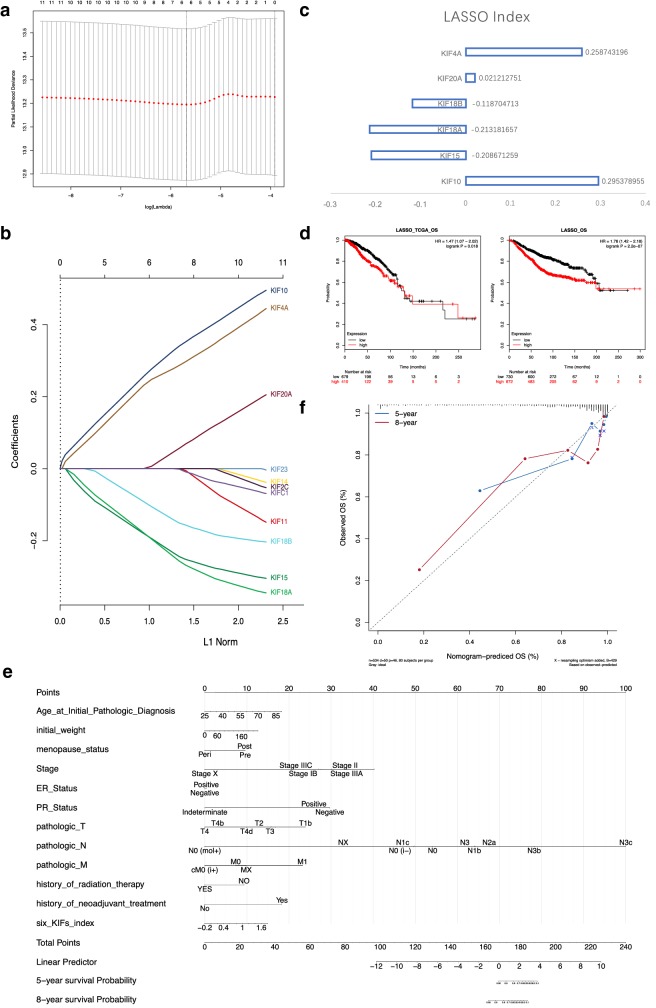
LASSO regression of overall survival-related KIFs and nomogram constructed. **a** Cvfit plot for the selection of LASSO regression lambda value as best cutoff. **b** LASSO regression of overall survival-related KIFs (KIF10, KIF11, KIF14, KIF15, KIF18A, KIF18B, KIF20A, KIF23, KIF2C, KIF4A, KIFC1). **c** Six-KIFs-based risk score generated by LASSO regression. **d** Validation of prognostic value of the six-KIFs-based risk score generated by LASSO regression using survival analyses with both TCGA-BRCA and combined chip-seq data. **e** Nomogram constructed with basic information of patients, pathologic information, clinical information and 6-KIFs-based risk score for prediction of 5/8-year overall survival of breast cancer patients. **f** Calibration plot done to self-validate the predictive efficacy of nomogram constructed

Given the expression profile and survival analyses of all KIFs, 11 overexpressed KIFs (KIF10, KIF11, KIF14, KIF15, KIF18A, KIF18B, KIF20A, KIF23, KIF2C, KIF4A, KIFC1) that demonstrated significant prognostic value in breast cancer were enrolled for LASSO regression to construct a KIFs-based risk score for prediction of OS in breast cancer. According to the cvfit plot, the minimal of lambda value was seen in 6, indicating a 6-KIFs-based risk score model the best for both accuracy and simplicity (Fig. 3a). Therefore, only KIF10, KIF15, KIF18A, KIF18B, KIF20A, KIF4A were included (Fig. 3b) and a 6-KIFs-based risk score was generated as below:

$$ \begin{aligned} {\text{Rs}} & = 0.30{\text{Exp}}\left( {{\text{KIF}}10} \right) - 0.21{\text{Exp}}\left( {{\text{KIF}}15} \right) - 0.21{\text{Exp}}\left( {{\text{KIF}}18{\text{A}}} \right) \hfill \\ & \quad - 0.12{\text{Exp}}\left( {{\text{KIF}}18{\text{B}}} \right) + 0.02{\text{Exp}}\left( {{\text{KIF}}20{\text{A}}} \right) + 0.26{\text{Exp}}\left( {{\text{KIF}}4{\text{A}}} \right) \hfill \\ \end{aligned} $$

Rs: risk score; Exp(X): the expression level of gene X; specific LASSO indexes were shown in Fig. 3c.

Validations of the 6-KIFs-based risk score done by Kaplan–Meier plots demonstrated good prognostic value in predicting OS of breast cancer, with higher risk score significantly correlated with worse outcomes in both TCGA-BRCA data [HR = 1.47 (1.07–2.02), logrank P = 0.018] and chip-seq data from GEO and METABRIC [HR = 1.76 (1.42–2.18), logrank P = 2.2e−07] (Fig. 3d).

### Nomogram

For the purpose of predicting OS in breast cancer patients, we conducted multivariate survival analyses to select KIFs-related clinical factors that can be enrolled to construct an accurate and stable nomogram. Comprehensive analyses were done focusing on 6 KIFs (KIF10, KIF15, KIF18A, KIF18B, KIF20A, KIF4A) identified by LASSO regression regarding RFS, OS and DMFS (Additional file 5). Factors enrolled in the nomogram include basic information of patients (age at initial pathologic diagnosis, initial weight, menopause status), pathologic information (stage, ER, PR status, TNM stage), clinical information (history of radiation therapy, history of neoadjuvant therapy) and 6-KIFs-based risk score (Fig. 3e). The lymph node status weighted the most in all factors, with N0 scores 0 while 100 for N3c. Menopause, ER and PR status showing a nuance of influence on the score, nevertheless, played an important role in maintaining the stability of the model, therefore, were included for better stability. The 6-KIFs-based risk score generated from LASSO analysis maintained a moderate influence on the total points, indicating the putative prognostic value in predicting the OS of breast cancer. Only self-validation was conducted using a calibration plot to evaluate the accuracy of the model and good accuracies were seen in both 5-year and 8-year survival prediction (Fig. 3f).

### mRNA and protein expression of KIFs are upregulated in breast cancer patients

**Fig. 4:**
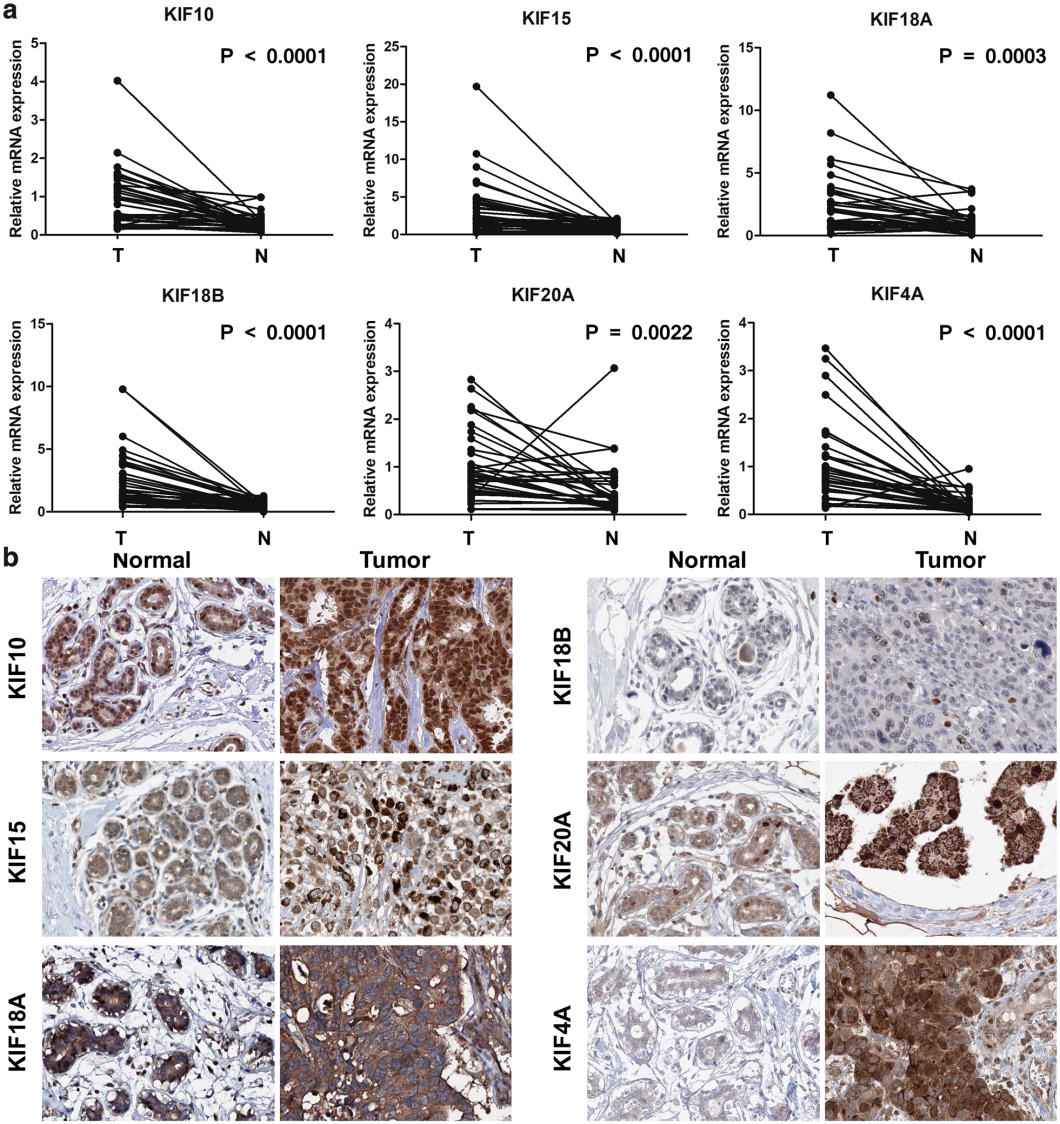
mRNA and protein expression of LASSO regression-selected KIFs in breast cancer patients. **a** Quantitative real-time PCR results of six LASSO regression-selected KIFs using samples of breast cancer patients. **b** Immunohistochemistry images of six LASSO regression-selected KIFs from TCGA breast cancer patients and GTEx normal breast tissue

A total of 30 pairs of samples were collected from breast cancer patients newly diagnosed and operated in the breast disease center of the first affiliated hospital of Sun Yat-sen University from March to April 2019. Patients enrolled all met the inclusion criteria described previously, with pathological stage II or III breast cancer diagnosis. Detailed patients’ characters were summarized in Additional file 6. All 6 KIFs selected by LASSO regression were seen overexpressed in tumor samples comparing to normal samples (KIF10, KIF15, KIF18B, KIF4A: P < 0.0001; KIF18A: P = 0.0003; KIF20A: P = 0.0022), which in accordance with bioinformatics results (Fig. 4a).

Immunocytochemistry showed KIF10, KIF15, KIF18A, KIF18B mainly localized to the cytosol and microtubules, besides, localized to the nucleoplasm in normal breast tissue. However, KIF20A, KIF4A mainly localized to the nucleoplasm, additional localization was seen in the cytokinetic bridge. Immunohistochemistry of TCGA breast cancer patients and GTEx normal breast tissue revealed protein level of 6 KIFs significantly upregulated in tumor samples despise of location (Fig. 4b). Antibody selected for each gene kept identical for better comparison. Quantity of samples selected remained above 75%, however, the staining and intensity in normal tissues, including adipocytes, glandular cells or myoepithelial cells, showed low or medium level, while high level of staining and strong intensity were seen in tumor samples. The notable differences observed from immunohistochemistry indicate putative prognostic value of KIFs as biomarkers for breast cancer.

### MSX1 identified as transcription factors of KIFs in breast cancer

**Fig. 5:**
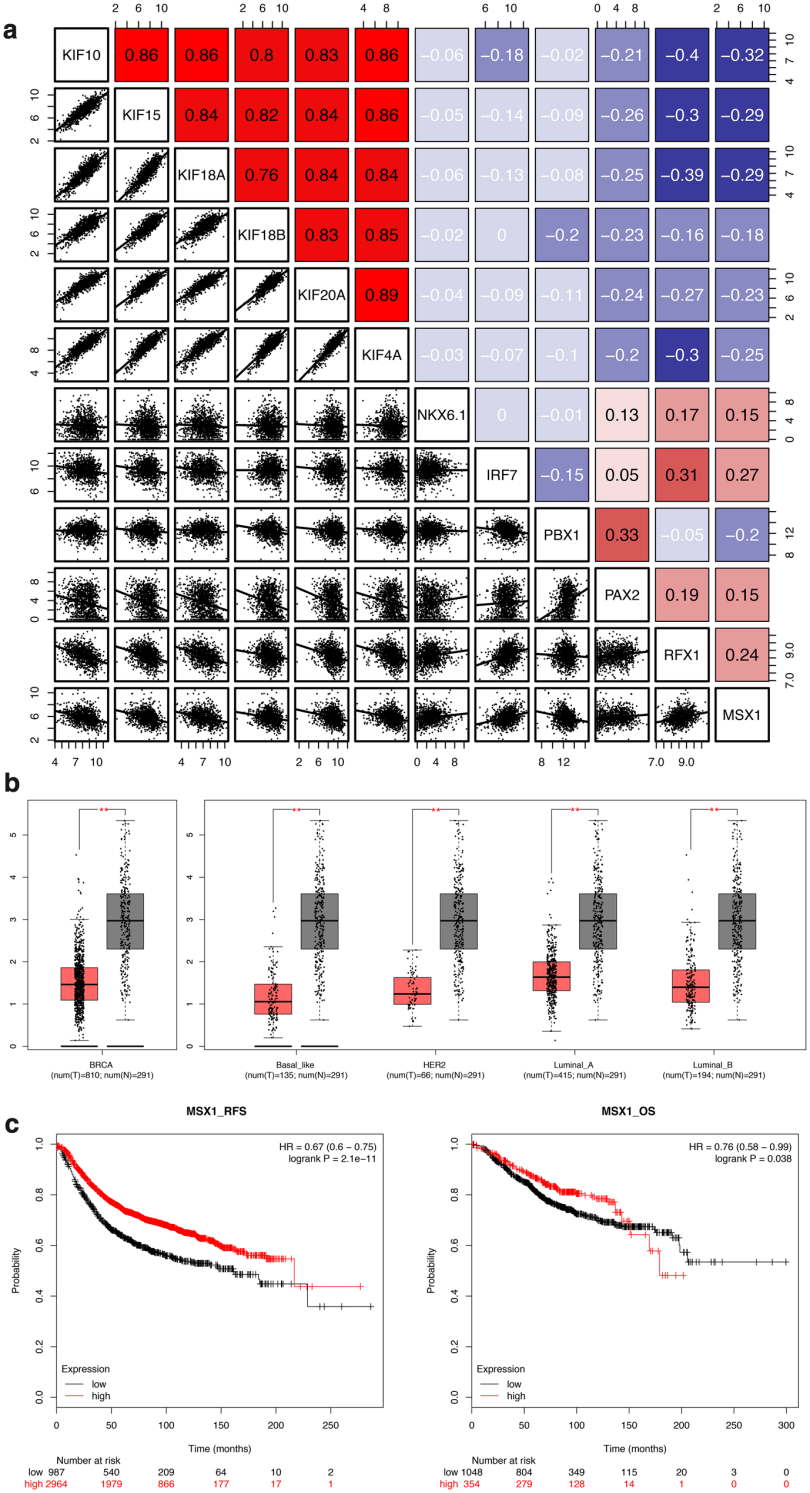
Transcription factors of LASSO regression-selected KIFs enriched. **a** Correlation plot of LASSO regression-selected KIFs and transcription factors enriched (NKX6.1, IRF7, PBX1, PAX2, RFX1, MSX1). **b** Expression of MSX1 between tumor and normal tissues in both breast cancer patients and intrinsic subtypes of breast cancer using both TCGA-BRCA and matched GTEx-breast normal data (Tumor = 810; Luminal A = 415; Luminal B = 194; HER2 = 66; Basal like = 135; Normal = 291). **c** Survival analyses of MSX1 regarding both RFS and OS using combined chip-seq data

In order to explore the upstream regulation mechanism of the 6 KIFs selected by LASSO regression, bioinformatics enrichment was done for putative transcription factors that regulate their expression. A total of 6 transcription factors (NKX6.1, IRF7, PBX1, PAX2, RFX1, MSX1) were found and all showed negative correlations with the 6 KIFs (Fig. 5a). Among 6 transcription factors enriched, PAX2, RFX1 and MSX1 showed stronger correlation than others, while expression profile and survival analyses revealed MSX1 significantly downregulated in breast cancer comparing to normal samples (Fig. 5b, P < 0.001) and high expression of MSX1 indicating better survival outcomes of breast cancer regarding both RFS [HR = 0 67 (0 6–0 75), logrank P = 2.1e−11] and OS [HR = 0 76 (0 58–0 99), logrank P = 0 038] (Fig. 5c). Therefore, we hypothesize that MSX1 works as a transcription factor of KIFs and decreased expression of MSX1 leads to the overexpression of KIFs, which contribute to the initiation, development and progress of breast cancer and indicate worse outcomes in breast cancer prognosis.

### GO and KEGG enrichments

**Fig. 6:**
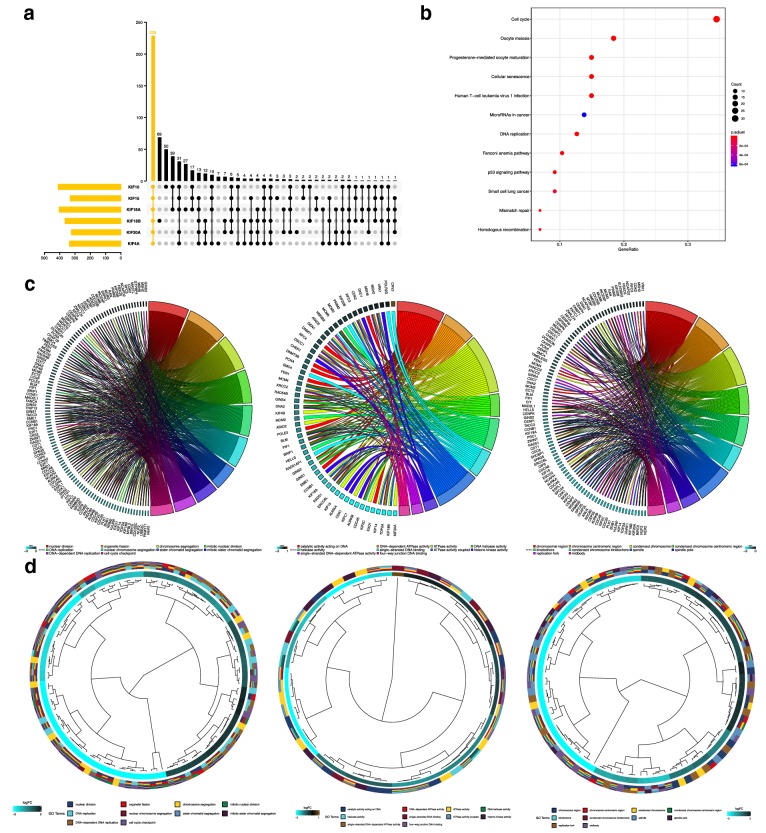
GO and KEGG enrichments of LASSO regression-selected KIFs. **a** Upset plot showing the intersection of co-expressed genes of the six LASSO regression-selected KIFs. **b** KEGG pathway enrichment of the six LASSO regression-selected KIFs. **c**, **d** GO function enrichment of the six LASSO regression-selected KIFs

For the sake of investigating mutually affected functions and downstream pathways of 6 overexpressed KIFs in breast cancer, we enriched and intersected co-expression genes of 6 KIFs. 229 intersecting genes were used for GO and KEGG enrichments (Fig. 6a). KEGG pathways enriched including cell cycle, oocyte meiosis, progesterone-mediated oocyte maturation, cellular senescence, human T-cell leukemia virus 1 infection, microRNAs in cancer, DNA replication, Fanconi anemia pathway and p53 signaling pathway (Fig. 6b). While GO enrichment found the 6 KIFs mainly functioned in nuclear division, DNA replication, chromosome segregation, mitotic nuclear division, catalytic activity acting on DNA, DNA-dependent ATPase activity, histone kinase activity, indicating important roles in chromosomal related activity in both biology and pathology (Fig. 6c, d; Additional file 7).

## Discussion

Kinesin superfamily has a long-reported significant influence on the initiation, development and progress of breast cancer [15, 16, 20, 21, 25, 26]. However, the prognostic value of whole family members was poorly done. Therefore, comprehensive bioinformatics analyses were done in our study using data from multi-dataset to explore the prognostic value, as well as regulatory mechanism, functions and putative pathways, of kinesin superfamily. A total of 20 differentially expressed KIFs were identified between breast cancer and normal tissue with 4 downregulated and 16 overexpressed. Survival analyses revealed 11 overexpressed KIFs (KIF10, KIF11, KIF14, KIF15, KIF18A, KIF18B, KIF20A, KIF23, KIF2C, KIF4A, KIFC1) significantly correlated with worse OS, RFS and DMFS of breast cancer, indicating efficient biomarkers for predicting the prognosis of breast cancer. Further analyses were done with a 6-KIFs-based risk score generated by LASSO regression, a nomogram was constructed with elements selected by multivariate survival analysis and an accurate predictive efficacy was validated. Expression profiles of the 6 KIFs selected (KIF10, KIF15, KIF18A, KIF18B, KIF20A, KIF4A) were testified by quantitative RT-PCR and immunohistochemistry. Overexpression was seen in all 6 KIFs in both mRNA and protein levels, which agrees with bioinformatics analyses, demonstrating stable and significant upregulation in breast cancer. Enrichments of regulatory mechanism revealed MSX1 a putative transcription factor that negatively regulates KIFs expression in breast cancer. GO and KEGG analyses were also done to explore mutual functions and pathways of KIFs in breast cancer.

Given the results done in our study, KIFs were demonstrated solid prognostic value with significantly differential expressions and strong correlations with the survival of breast cancer by bioinformatics analyses and further quantitative RT-PCR and immunohistochemistry of patient samples also demonstrated a significant difference between cancer and normal tissue, indicating putative efficacy as biomarkers for breast cancer. Previous work is done by Song et al. using only TCGA data found 21 significantly differential-expressed KIFs, among which just KIF4A was further identified as OS-related, while overexpression of KIF15, KIF20A, KIF23, KIF2C related to OS after adjusted for tumor stage and age [57]. Comparing to the results given in our study, similar expression profiles were seen with an extension of normal samples from GTEx. However, by using combined data from multi-dataset, the significant prognostic value was seen in most KIFs regarding either OS, RFS or DMFS. Furthermore, survival analyses were done with only TCGA data also showed significant correlations between the expression of KIFs and survival outcomes of breast cancer, OS and RFS. Given the purpose of exploring the prognostic value of KIFs, best cutoffs were used for grouping instead of median expressions, meanwhile, larger samples ensured better accuracy and sensitivity. Additionally, validations made with operating samples from breast cancer patients in both mRNA and protein levels further supported KIFs working as executable clinical biomarkers. Therefore, our study demonstrated greater efficacy and availability of KIFs in predicting breast cancer prognosis.

The 6 KIFs selected by LASSO regression in our study were demonstrated significant prognostic value with overexpression strongly correlated with worse outcomes in breast cancer, not only overall survival but also relapse and distant metastasis, by bioinformatics analyses. Validations can be made from studies published, focusing on the biological and tumorigenic mechanism of KIFs. Previously reported biological functions of KIFs mainly involved in the regulation of mitosis [58]. During prophase to prometaphase transition, KIF15 works as an interaction partner Ki67 and is required for spindle elongation and the maintenance of spindle bipolarity [59, 60]. KIF10, KIF18A and KIF18B are reported to be essential to the progression from metaphase to anaphase with different functions [61, 62]. KIF10 mainly participates in microtubule–kinetochore capture and mitotic checkpoint signaling, therefore plays an important role in chromosome congression and alignment [61, 62], while KIF18A and KIF18B, two related members of kinesin-8 family, both regulate microtubule dynamics at the plus end, controlling correct chromosome positioning and the length of astral microtubules, respectively [63–65]. KIF20A was reported to be functioning during cytokinesis by regulating furrow ingression and several other events that are essential for successful cytokinesis [66, 67]. KIF4A, among all six KIFs selected in our study, is the only one that functionally involved in multi-stages of mitosis, participating in chromosome condensation, anaphase spindle mid-zone formation and cytokinesis [50, 62, 68]. Given the hyperactive proliferation of tumor cells, overexpression of the six KIFs selected as expected, which in accordance with the results given in our study, and further demonstrations were found on both cellular and molecular levels reported in previous studies [69–72]. Tumorigenic functions of KIFs affect various aspects of breast cancer, including metastasis, progression and chemotherapy resistance. Silencing of KIF10 and KIF18A were both reported inhibitions to the proliferation of breast cancer cells via deregulating cell division [20, 69]. Lysosomal stability was demonstrated to enhance the survival of breast cancer cells while the knocking down of KIF20A conduced the permeabilization of the lysosomal membrane, which in turn, causing cellular death [73]. KIF18A, KIF15 and KIF4A were demonstrated prognostic biomarkers for prediction of clinical outcomes [20, 70]. Furthermore, expression of KIF18A was associated with cancer grade and metastasis status and may facilitate cancer cell migration by deregulating microtubule stability [20]. Given both biological and tumorigenic functions of the six KIFs selected, which is highly consistent with our results from bioinformatics analyses, the prognostic value of six KIFs was seen in predicting clinical outcomes of breast cancer patients with high expression of KIFs highly correlated with worse survival endings, including overall survival, relapse-free survival and distant metastasis-free survival.

Numerous works have been done focusing on the exploration and validation of breast cancer biomarkers for better clinical stratification of patients and more efficacious treatment. Early predictive models generated from clinical data and SEER database with only clinical factors had been demonstrated a lack of efficacy in the surge of sequencing technology. Recent days have seen models using multi-omics data to pursue better accuracy but failed in clinical transformation owing to the limitation of detection technology and standardized criteria. Meanwhile, several multi-gene test panels had already validated good utility by randomized clinical trials and been recommended by NCCN guidelines like the 21-gene test [74], MammaPrint [75] or PAM50 [76]. A within-patient comparison had been done by Ivana Sestak et al. between multiple molecular signatures that are available for managing ER-positive, ERBB2-negative breast cancer after 5-year endocrine therapy in the TransATAC cohort. The signatures providing the most prognostic information were the PAM50 (hazard ratio [HR], 2.56; 95% CI 1.96–3.35), followed by the Breast Cancer Index (HR, 2.46; 95% CI 1.88–3.23) and EndoPredict (HR, 2.14; 95% CI 1.71–2.68). Each provided significantly more information than the Clinical Treatment Score (HR, 1.99; 95% CI 1.58–2.50), the 21-gene score (HR, 1.69; 95% CI 1.40–2.03), and the 4-marker immunohistochemical score (HR, 1.95; 95% CI 1.55–2.45) [77]. These results demonstrated better efficacy of predictive models combined with molecular and clinical information than each alone. Given the essential roles of KIFs in breast cancer, a comprehensive analysis combining molecular expression and clinical features has been done in our study tying to highlight the prognostic potency of a six-KIFs score based predictive model. Despise a lack of large cohort comparison with any other biomarkers, instead, we validated good efficacy as well as a clinical utility by qPCR and IHC which are easy-access and standardized methods.

Although the KIF family has been shown to play an essential role in various aspects of breast cancer, the development of drugs targeting KIFs has not been satisfactory. Previously reported KIFs-targeting drugs including GSK923295 (a KIF10 inhibitor) [78], Quinazolinedione and phthalimide inhibitors (both KIF15 inhibitors) [79], BTB1 (an inhibitor of KIF18A) [80], Paprotrain (the first known inhibitor of MKLP2) [81]. However, no clinical trials were done in breast cancer and a ‘double-edged sword’ effect was seen in the therapeutic efficacy of the KIF10 inhibitor [82], indicating an unclear treatment window. The limitation shown in drug development raised controversy in the clinical significance of kinesin superfamily. Furthermore, analyses found regulatory correlations between members, with KIF10 regulated by KIF18A [83], which indicates a putative deficiency in singling out any KIFs to analyze alone rather than balancing the interplay between tumor-related KIFs [84]. Therefore, our analyses combined all KIFs to explore the prognostic value and putative regulatory mechanism of KIFs. Despise the correlations between KIFs and the prognosis of breast cancer, a putative transcription factor MSX1 was identified as a repressive upstream with a significant under-expression in breast cancer, which may lead to the overexpression of KIFs and further contribute to the initiation, progression and prognosis of breast cancer. This may give a new perspective into the therapeutic value of KIFs by revealing a putative mutual regulator which significantly affects the expression of tumor-related KIFs, therefore, may serve as a potential drug target by influencing kinesin superfamily.

MSX1, a member of the muscle segment homeobox gene family, was long identified as a transcriptional repressor during various biological processes [85]. The essential roles of MSX1 were demonstrated in multiple malignancies. High-throughput global expression profiling of lung cancer cells revealed promoter methylation of MSX1 a novel biomarker for primary lung, breast, colon, and prostate cancers [86]. Cellular experiments validated hypomethylation of CpG sites within the MSX1 gene highly associated with resistant high-grade serous ovarian cancer (HGSOC) disease at presentation and identified expression of MSX1 as conferring platinum drug sensitivity [87]. By interacting with P53 tumor suppressor, MSX1 was demonstrated as an inhibitor to tumor growth as well as an inducer to cancer cell apoptosis [88]. From our bioinformatics enrichments of KIFs, MSX1 showed potency in functioning as a therapeutic target for breast cancer treatment by generally repressing the expression of survival-related KIFs, which may need further tests in both pharmaceutical development and clinical trials.

KEGG analyses found various putative downstream pathways affected by the alteration of KIFs, among which human T-cell leukemia virus 1 infection pathway indicating potential correlations between KIFs and immunity. Researches published demonstrated the correlation predicted by our bioinformatics analyses. KIF7 was reported to be required for T-cell development with the deficiency of KIF7 leading to the increase of premature CD44+CD25+CD4−CD8− thymocyte progenitor population while a decrease of differentiated CD4+CD8+ double- positive (DP) cell [89]. Furthermore, KIF20A-derived long peptides were identified bearing naturally processed epitopes recognized by CD4(+) T cells and CTLs, which induce tumor-specific T-helper type 1 (TH1) cells and CTLs in head-and-neck malignant tumor tissues [11]. Other pathways enriched include Fanconi anemia pathway and p53 signaling pathway. Previously published studies validated repressed expression of KIF2C regulated by P53 via down-regulation of Sp1 level in human tumor cells [90], however, no report was found focusing on Fanconi anemia and KIFs, which need further exploration.

In conclusion, our study demonstrated the significant overexpression of tumor-related KIFs by bioinformatics analyses, which correlate with worse outcomes of breast cancer patients, therefore may work as prognostic biomarkers. A nomogram containing LASSO-generated six-KIFs-index was generated and validated a good prediction efficacy. Further analyses revealed MSX1 a putative transcription factor that negatively regulates the expression of KIFs in breast cancer and may work as a putative drug target.

## Supplementary information

**Additional file 1.** Primer pairs for 6 KIFs included in LASSO index.

**Additional file 2.** Subtype expression profiles of kinesin superfamily in breast cancer. Expression comparisons of 20 significantly differential-expressed KIFs (KIF17, KIF26A, KIF7, KIFC3, KIF10, KIF11, KIF14, KIF15, KIF18A, KIF18B, KIF20A, KIF20B, KIF22, KIF23, KIF24, KIF26B, KIF2C, KIF3B, KIF4A, KIFC1) within subtypes using both TCGA-BRCA and matched GTEx-breast normal data (Tumor = 810; Normal = 291). *P < 0.05; **P < 0.001.

**Additional file 3.** Comparisons of subtype expression profiles of kinesin superfamily in breast cancer. Expression comparisons of 20 significantly differential-expressed KIFs (KIF17, KIF26A, KIF7, KIFC3, KIF10, KIF11, KIF14, KIF15, KIF18A, KIF18B, KIF20A, KIF20B, KIF22, KIF23, KIF24, KIF26B, KIF2C, KIF3B, KIF4A, KIFC1) among subtypes using TCGA-BRCA data. *P < 0.05; **P < 0.001.

**Additional file 4.** Survival analyses of kinesin superfamily using TCGA-BRCA data. Survival analyses of 18 significantly differential-expressed KIFs (KIF26A, KIF7, KIFC3, KIF10, KIF11, KIF14, KIF15, KIF18A, KIF18B, KIF20A, KIF20B, KIF22, KIF23, KIF24, KIF26B, KIF2C, KIF3B, KIFC1) in breast cancer regarding both OS and RFS using TCGA data. Red: high expression group; black: low expression group.

**Additional file 5.** Multivariate survival analysis of RFS, OS and DMFS focusing on 6 KIFs related clinical factors.

**Additional file 6.** Clinical characters of patients enrolled.

**Additional file 7.** (1) GO enrichment results of the 6 KIFs selected by LASSO regression. (2) KEGG enrichment results of the 6 KIFs selected by LASSO regression.

## Data Availability

The datasets generated and/or analysed during the current study are available in the UCSC XENA repository, [https://tcga.xenahubs.net]. Data used included the Cancer Genome Atlas (TCGA, http://can-cergenome.nih.gov/), the GTEx projects, Gene Expression Omnibus (GEO, https://www.ncbi.nlm.nih.gov/ geo/) and Molecular Taxonomy of Breast Cancer International Consortium (METABRIC) project.

## Abbreviations

KIFs: Kinesin superfamily
TCGA: The cancer genome atlas
GEO: Gene expression omnibus
METABRIC: Molecular Taxonomy of Breast Cancer International Consortium
RFS: Relapse-free survival
OS: Overall survival
DMFS: Distant metastasis-free survival
GO: Gene oncology
KEGG: Kyoto Encyclopedia of Genes and Genomes
MSX1: Msh Homeobox 1
ATP: Adenosine triphosphate
