# Supplementary material for: Overexpression of kinesin superfamily members as prognostic biomarkers of breast cancer
Source: Cancer Cell Int. 2020 Apr 15;20:123. doi: 10.1186/s12935-020-01191-1 (PMC7161125; doi:10.1186/s12935-020-01191-1)
Supplement: Supplementary file 1 — Additional file 1. Primer pairs for 6 KIFs included in LASSO index. [file 12935_2020_1191_MOESM1_ESM.docx]

**Additional Materials**

**Title: Overexpression of kinesin superfamily members as prognostic biomarkers of breast cancer.**

Additional file 1: primer pairs for 6 KIFs included in LASSO index.

| primer | sequence |
| --- | --- |
| GAPDH-F | GCACCGTCAAGGCTGAGAAC |
| GAPDH-R | TGGTGAAGACGCCAGTGGA |
| KIF4A-F | TACTGCGGTGGAGCAAGAAG |
| KIF4A-R | CATCTGCGCTTGACGGAGAG |
| KIF10-F | GATTCTGCCATACAAGGCTACAA |
| KIF10-R | TGCCCTGGGTATAACTCCCAA |
| KIF15-F | AGGAATCTGTATTCGCAACTGTG |
| KIF15-R | ACTTCGTGGGATTACTCCTCTC |
| KIF18A-F | TGGACTTACTTTACACCAGCCC |
| KIF18A-R | GCTGTTTTGTCTTGTTGTCGC |
| KIF18B-F | TACGAGGACACGTACAACACC |
| KIF18B-R | CAGGCTGGTCACATTGCTC |
| KIF20A-F | TTGAGGGTTAGGCCCTTGTTA |
| KIF20A-R | GTCCTTGGGTGCTTGTAGAAC |
