# Supplementary material for: Overexpression of kinesin superfamily members as prognostic biomarkers of breast cancer
Source: Cancer Cell Int. 2020 Apr 15;20:123. doi: 10.1186/s12935-020-01191-1 (PMC7161125; doi:10.1186/s12935-020-01191-1)
Supplement: Supplementary file 3 — Additional file 3. Comparisons of subtype expression profiles of kinesin superfamily in breast cancer. Expression comparisons of 20 significantly differential-expressed KIFs (KIF17, KIF26A, KIF7, KIFC3, KIF10, KIF11, KIF14, KIF15, KIF18A, KIF18B, KIF20A, KIF20B, KIF22, KIF23, KIF24, KIF26B, KIF2C, KIF3B, KIF4A, KIFC1) among subtypes using TCGA-BRCA data. *P < 0.05; **P < 0.001. [file 12935_2020_1191_MOESM3_ESM.docx]

**Additional file 3: Comparisons of subtype expression profiles of kinesin superfamily in breast cancer.** Expression comparisons of 20 significantly differential-expressed KIFs (KIF17, KIF26A, KIF7, KIFC3, KIF10, KIF11, KIF14, KIF15, KIF18A, KIF18B, KIF20A, KIF20B, KIF22, KIF23, KIF24, KIF26B, KIF2C, KIF3B, KIF4A, KIFC1) among subtypes using TCGA-BRCA data. * P < 0.05; ** P < 0.001.
