# Supplementary material for: Overexpression of kinesin superfamily members as prognostic biomarkers of breast cancer
Source: Cancer Cell Int. 2020 Apr 15;20:123. doi: 10.1186/s12935-020-01191-1 (PMC7161125; doi:10.1186/s12935-020-01191-1)
Supplement: Supplementary file 4 — Additional file 4. Survival analyses of kinesin superfamily using TCGA-BRCA data. Survival analyses of 18 significantly differential-expressed KIFs (KIF26A, KIF7, KIFC3, KIF10, KIF11, KIF14, KIF15, KIF18A, KIF18B, KIF20A, KIF20B, KIF22, KIF23, KIF24, KIF26B, KIF2C, KIF3B, KIFC1) in breast cancer regarding both OS and RFS using TCGA data. Red: high expression group; black: low expression group. [file 12935_2020_1191_MOESM4_ESM.docx]

**Additional file 4: Survival analyses of kinesin superfamily using TCGA-BRCA data.** Survival analyses of 18 significantly differential-expressed KIFs (KIF26A, KIF7, KIFC3, KIF10, KIF11, KIF14, KIF15, KIF18A, KIF18B, KIF20A, KIF20B, KIF22, KIF23, KIF24, KIF26B, KIF2C, KIF3B, KIFC1) in breast cancer regarding both OS and RFS using TCGA data. Red: high expression group; black: low expression group.
