# Supplementary material for: Overexpression of kinesin superfamily members as prognostic biomarkers of breast cancer
Source: Cancer Cell Int. 2020 Apr 15;20:123. doi: 10.1186/s12935-020-01191-1 (PMC7161125; doi:10.1186/s12935-020-01191-1)
Supplement: Supplementary file 5 — Additional file 5. Multivariate survival analysis of RFS, OS and DMFS focusing on 6 KIFs related clinical factors. [file 12935_2020_1191_MOESM5_ESM.docx]

**Additional file** **5: Multivariate survival analysis of RFS, OS and DMFS focusing on 6 KIFs related clinical factors.**

|  | | KIF10 | | KIF15 | | KIF18A | | KIF18B | | KIF20A | | KIF4A | |
| --- | --- | --- | --- | --- | --- | --- | --- | --- | --- | --- | --- | --- | --- |
|  |  | HR (95% CI) | logrank P | HR (95% CI) | logrank P | HR (95% CI) | logrank P | HR (95% CI) | logrank P | HR (95% CI) | logrank P | HR (95% CI) | logrank P |
| **RFS** | | 2.27 (1.95 − 2.63) | <0.0001 | 1.67 (1.48 − 1.88) | <0.0001 | 1.91 (1.65 − 2.2) | <0.0001 | 1.46 (1.3 − 1.63) | <0.0001 | 2.01 (1.74 − 2.33) | <0.0001 | 2.17 (1.86 − 2.52) | <0.0001 |
| ER status | |  |  |  |  |  |  |  |  |  |  |  |  |
|  | ER positive | 2.02 (1.66 − 2.44) | <0.0001 | 1.73 (1.46 − 2.06) | <0.0001 | 1.83 (1.55 − 2.15) | <0.0001 | 1.87 (1.56 − 2.23) | <0.0001 | 2.06 (1.74 − 2.45) | <0.0001 | 2.04 (1.71 − 2.45) | <0.0001 |
|  | ER negative | 1.36 (1.06 − 1.73) | 0.014 | 1.33 (1.1 − 1.7) | 0.011 | 1.19 (0.94 − 1.5) | 0.140 | 1.12 (0.88 − 1.4) | 0.370 | 1.15 (0.9 − 1.5) | 0.260 | 1.33 (1.1 − 1.7) | 0.018 |
| PR status | |  |  |  |  |  |  |  |  |  |  |  |  |
|  | PR positive | 2.67 (1.62 − 4.4) | <0.0001 | 2.42 (1.64 − 3.58) | <0.0001 | 1.92 (1.31 − 2.81) | 0.001 | 3.42 (1.96 − 5.96) | <0.0001 | 2.92 (1.89 − 4.53) | <0.0001 | 2.63 (1.81 − 3.83) | <0.0001 |
|  | PR negative | 1.58 (1.17 − 2.14) | 0.003 | 1.36 (0.99 − 1.88) | 0.061 | 1.19 (0.89 − 1.6) | 0.240 | 1.4 (1.03 − 1.92) | 0.032 | 1.66 (1.15 − 2.39) | 0.006 | 1.29 (0.96 − 1.74) | 0.087 |
| HER2 status | |  |  |  |  |  |  |  |  |  |  |  |  |
|  | HER2 positive | 1.32 (0.79 − 2.18) | 0.290 | 1.52 (0.98 − 2.4) | 0.058 | 1.45 (0.93 − 2.3) | 0.095 | 1.25 (0.77 − 2.02) | 0.370 | 1.25 (0.79 − 2) | 0.340 | 1.54 (0.99 − 2.4) | 0.054 |
|  | HER2 negative | 2.18 (1.67 − 2.85) | <0.0001 | 2.28 (1.62 − 3.2) | <0.0001 | 1.92 (1.47 − 2.51) | <0.0001 | 2.23 (1.71 − 2.9) | <0.0001 | 2.56 (1.88 − 3.49) | <0.0001 | 2.65 (1.89 − 3.72) | <0.0001 |
| Lymph node status | |  |  |  |  |  |  |  |  |  |  |  |  |
|  | Lymph node positive | 1.64 (1.35 − 2.01) | <0.0001 | 1.82 (1.43 − 2.31) | <0.0001 | 1.79 (1.45 − 2.21) | <0.0001 | 2.03 (1.58 − 2.61) | <0.0001 | 1.95 (1.58 − 2.41) | <0.0001 | 2.17 (1.72 − 2.74) | <0.0001 |
|  | Lymph node negative | 1.82 (1.53 − 2.17) | <0.0001 | 1.63 (1.37 − 1.93) | <0.0001 | 1.64 (1.39 − 1.95) | <0.0001 | 1.76 (1.48 − 2.1) | <0.0001 | 2.02 (1.67 − 2.45) | <0.0001 | 2.05 (1.65 − 2.54) | <0.0001 |
| Grade | |  |  |  |  |  |  |  |  |  |  |  |  |
|  | I | 2.26 (1.35 − 3.8) | 0.002 | 2.32 (1.05 − 5.12) | 0.032 | 2.77 (1.64 − 4.69) | <0.0001 | 2.35 (1.39 − 3.97) | 0.001 | 3.57 (2.12 − 6.03) | <0.0001 | 3.61 (2.14 − 6.09) | <0.0001 |
|  | II | 1.93 (1.51 − 2.46) | <0.0001 | 1.84 (1.43 − 2.36) | <0.0001 | 1.75 (1.37 − 2.22) | <0.0001 | 1.74 (1.37 − 2.22) | <0.0001 | 1.82 (1.42 − 2.33) | <0.0001 | 1.76 (1.38 − 2.25) | <0.0001 |
|  | III | 1.31 (1.02 − 1.67) | 0.031 | 1.3 (1.02 − 1.66) | 0.031 | 1.11 (0.89 − 1.38) | 0.350 | 1.19 (0.96 − 1.48) | 0.120 | 1.13 (0.89 − 1.43) | 0.300 | 1.15 (0.9 − 1.47) | 0.260 |
| Intrinsic subtype | |  |  |  |  |  |  |  |  |  |  |  |  |
|  | Luminal A | 2.3 (1.88 − 2.81) | <0.0001 | 1.75 (1.47 − 2.07) | <0.0001 | 2.01 (1.69 − 2.38) | <0.0001 | 1.62 (1.37 − 1.92) | <0.0001 | 1.88 (1.59 − 2.23) | <0.0001 | 2.03 (1.71 − 2.41) | <0.0001 |
|  | Luminal B | 1.69 (1.33 − 2.14) | <0.0001 | 1.56 (1.27 − 1.91) | <0.0001 | 1.41 (1.16 − 1.7) | 0.000 | 1.29 (1.06 − 1.56) | 0.011 | 1.44 (1.19 − 1.75) | 0.000 | 1.42 (1.17 − 1.72) | 0.000 |
|  | HER-2 | 1.62 (1.08 − 2.42) | 0.018 | 1.55 (1.03 − 2.34) | 0.036 | 1.27 (0.86 − 1.9) | 0.240 | 1.72 (1.1 − 2.6) | 0.009 | 1.26 (0.84 − 1.89) | 0.250 | 1.27 (0.83 − 1.9) | 0.270 |
|  | TNBC | 1.6 (1.24 − 2.07) | 0.000 | 1.41 (1.1 − 1.8) | 0.008 | 1.2 (0.91 − 1.6) | 0.190 | 1.59 (1.2 − 2.1) | 0.000 | 1.2 (0.9 − 1.6) | 0.210 | 1.2 (0.91 − 1.6) | 0.190 |
| TP53 status | |  |  |  |  |  |  |  |  |  |  |  |  |
|  | mutated | 1.39 (0.86 − 2.27) | 0.180 | 1.52 (0.95 − 2.5) | 0.082 | 1.92 (1 − 3.6) | 0.035 | 2.33 (1.2 − 4.4) | 0.009 | 1.52 (0.83 − 2.8) | 0.170 | 1.42 (0.88 − 2.28) | 0.150 |
|  | wild type | 2.77 (1.5 − 5.09) | 0.001 | 1.91 (1.24 − 2.95) | 0.003 | 1.98 (1.16 − 3.36) | 0.010 | 2.19 (1.42 − 3.39) | 0.000 | 2.87 (1.67 − 4.94) | <0.0001 | 1.88 (1.23 − 2.86) | 0.003 |
| Endocrine therapy | |  |  |  |  |  |  |  |  |  |  |  |  |
|  | Tamoxifen only | 2.35 (1.56 − 3.54) | <0.0001 | 1.75 (1.23 − 2.48) | 0.002 | 1.83 (1.36 − 2.46) | <0.0001 | 2.39 (1.69 − 3.37) | <0.0001 | 2.25 (1.66 − 3.06) | <0.0001 | 2 (1.44 − 2.78) | <0.0001 |
|  | any endocrine therapy | 2.15 (1.62 − 2.86) | <0.0001 | 1.68 (1.3 − 2.16) | <0.0001 | 1.99 (1.56 − 2.55) | <0.0001 | 2.27 (1.71 − 3.03) | <0.0001 | 2.25 (1.73 − 2.93) | <0.0001 | 2.23 (1.67 − 2.97) | <0.0001 |
|  | no endocrine therapy | 1.96 (1.61 − 2.39) | <0.0001 | 1.57 (1.29 − 1.91) | <0.0001 | 1.54 (1.29 − 1.84) | <0.0001 | 1.65 (1.38 − 1.96) | <0.0001 | 1.71 (1.4 − 2.08) | <0.0001 | 1.74 (1.43 − 2.12) | <0.0001 |
| Chemotherapy | |  |  |  |  |  |  |  |  |  |  |  |  |
|  | any chemotherapy | 1.82 (1.37 − 2.42) | <0.0001 | 1.32 (0.99 − 1.76) | 0.055 | 1.41 (1.08 − 1.84) | 0.012 | 1.52 (1.17 − 1.99) | 0.002 | 1.57 (1.14 − 2.16) | 0.006 | 1.7 (1.2 − 2.41) | 0.002 |
|  | adjuvant only | 1.93 (1.41 − 2.65) | <0.0001 | 1.34 (0.99 − 1.83) | 0.060 | 1.47 (1.08 − 2.01) | 0.015 | 1.7 (1.26 − 2.3) | 0.000 | 1.77 (1.23 − 2.56) | 0.002 | 1.62 (1.17 − 2.24) | 0.003 |
|  | neoadjuvant only | 1.6 (0.89 − 2.87) | 0.110 | 1.52 (0.87 − 2.7) | 0.140 | 1.43 (0.81 − 2.51) | 0.210 | 1.52 (0.85 − 2.7) | 0.150 | 1.56 (0.9 − 2.7) | 0.110 | 1.39 (0.76 − 2.55) | 0.280 |
|  | no chemotherapy | 2.21 (1.82 − 2.7) | <0.0001 | 1.72 (1.45 − 2.04) | <0.0001 | 1.78 (1.51 − 2.1) | <0.0001 | 1.94 (1.63 − 2.31) | <0.0001 | 2.04 (1.71 − 2.45) | <0.0001 | 2.17 (1.76 − 2.68) | <0.0001 |
|  |  |  |  |  |  |  |  |  |  |  |  |  |  |
| **OS** | | 1.96 (1.51 − 2.56) | <0.0001 | 1.6 (1.29 − 1.99) | <0.0001 | 2.46 (1.85 − 3.27) | <0.0001 | 2.16 (1.72 − 2.73) | <0.0001 | 2.37 (1.82 − 3.08) | <0.0001 | 2.11 (1.66 − 2.7) | <0.0001 |
| ER status | |  |  |  |  |  |  |  |  |  |  |  |  |
|  | ER positive | 2.25 (1.58 − 3.23) | <0.0001 | 2.15 (1.5 − 3.1) | <0.0001 | 3.12 (2.16 − 4.49) | <0.0001 | 3.44 (2.05 − 5.77) | <0.0001 | 2.9 (1.92 − 4.36) | <0.0001 | 2.8 (1.94 − 4.04) | <0.0001 |
|  | ER negative | 1.55 (0.85 − 2.82) | 0.150 | 0.75 (0.43 − 1.3) | 0.300 | 0.7 (0.43 − 1.13) | 0.140 | 1.59 (0.96 − 2.61) | 0.067 | 0.61 (0.38 − 0.96) | 0.031 | 0.53 (0.33 − 0.85) | 0.008 |
| PR status | |  |  |  |  |  |  |  |  |  |  |  |  |
|  | PR positive | 3.32 (0.87 − 12.64) | 0.063 | 2.75 (0.73 − 10.32) | 0.120 | 9.82 (1.98 − 48.7) | 0.001 | 0.52 (0.13 − 1.98) | 0.330 | 1.91 (0.51 − 7.12) | 0.330 | 4.22 (1.03 − 17.36) | 0.031 |
|  | PR negative | 2.11 (0.82 − 5.44) | 0.110 | 2.01 (0.79 − 5.08) | 0.130 | 2.46 (0.92 − 6.57) | 0.062 | 3.36 (1.2 − 9.44) | 0.015 | 2.88 (0.66 − 12.55) | 0.140 | 3.6 (0.83 − 15.64) | 0.068 |
| HER2 status | |  |  |  |  |  |  |  |  |  |  |  |  |
|  | HER2 positive | 0.66 (0.3 − 1.44) | 0.290 | 0.63 (0.31 − 1.28) | 0.200 | 1.8 (0.69 − 4.69) | 0.220 | 0.41 (0.14 − 1.17) | 0.086 | 0.4 (0.19 − 0.87) | 0.017 | 1.51 (0.71 − 3.21) | 0.280 |
|  | HER2 negative | 3.26 (1.26 − 8.44) | 0.010 | 4.01 (0.93 − 17.24) | 0.044 | 3.25 (1.31 − 8.05) | 0.007 | 7.06 (0.95 − 52.71) | 0.026 | 8.79 (1.18 − 65.5) | 0.010 | 2.72 (1 − 7.44) | 0.042 |
| Lymph node status | |  |  |  |  |  |  |  |  |  |  |  |  |
|  | Lymph node positive | 0.76 (0.48 − 1.22) | 0.260 | 1.51 (0.99 − 2.31) | 0.054 | 1.8 (1.11 − 2.92) | 0.015 | 1.65 (1.04 − 2.61) | 0.030 | 1.78 (1.11 − 2.85) | 0.016 | 1.69 (1.05 − 2.74) | 0.030 |
|  | Lymph node negative | 2.53 (1.7 − 3.75) | <0.0001 | 2.11 (1.41 − 3.15) | 0.000 | 2.42 (1.65 − 3.55) | <0.0001 | 2.21 (1.53 − 3.21) | <0.0001 | 3.05 (1.89 − 4.9) | <0.0001 | 3.78 (2.12 − 6.75) | <0.0001 |
| Grade | |  |  |  |  |  |  |  |  |  |  |  |  |
|  | I | 2.62 (1.04 − 6.6) | 0.034 | 0.52 (0.21 − 1.29) | 0.150 | 2.68 (1.11 − 6.5) | 0.023 | 2.75 (1.08 − 7.02) | 0.028 | 3.63 (1.43 − 9.21) | 0.004 | 3.2 (1.06 − 9.69) | 0.030 |
|  | II | 1.91 (1.23 − 2.96) | 0.003 | 2.87 (1.7 − 4.85) | <0.0001 | 2.73 (1.68 − 4.42) | <0.0001 | 2.11 (1.37 − 3.25) | 0.001 | 3.28 (1.94 − 5.55) | <0.0001 | 3.08 (1.8 − 5.26) | <0.0001 |
|  | III | 1.35 (0.91 − 2.01) | 0.130 | 0.75 (0.54 − 1.04) | 0.087 | 1.44 (0.97 − 2.14) | 0.067 | 0.72 (0.49 − 1.04) | 0.077 | 0.72 (0.52 − 1) | 0.046 | 0.72 (0.51 − 1.03) | 0.071 |
| Intrinsic subtype | |  |  |  |  |  |  |  |  |  |  |  |  |
|  | Luminal A | 2.43 (1.71 − 3.46) | <0.0001 | 2.28 (1.58 − 3.27) | <0.0001 | 2.63 (1.83 − 3.77) | <0.0001 | 2.76 (1.94 − 3.93) | <0.0001 | 2.4 (1.69 − 3.43) | <0.0001 | 2.74 (1.93 − 3.9) | <0.0001 |
|  | Luminal B | 1.44 (0.9 − 2.3) | 0.120 | 1.14 (0.77 − 1.68) | 0.510 | 1.82 (1.17 − 2.84) | 0.008 | 1.56 (1.03 − 2.37) | 0.036 | 1.97 (1.23 − 3.17) | 0.004 | 1.56 (0.97 − 2.51) | 0.064 |
|  | HER-2 | 1.6 (0.82 − 3.15) | 0.170 | 2.07 (1.06 − 4.03) | 0.028 | 0.74 (0.38 − 1.43) | 0.370 | 2.8 (1.46 − 5.38) | 0.001 | 2.13 (1.08 − 4.2) | 0.025 | 1.88 (0.95 − 3.69) | 0.064 |
|  | TNBC | 0.6 (0.37 − 0.99) | 0.042 | 0.51 (0.31 − 0.84) | 0.007 | 0.5 (0.28 − 0.9) | 0.018 | 0.35 (0.21 − 0.6) | <0.0001 | 0.38 (0.23 − 0.63) | <0.0001 | 0.45 (0.28 − 0.74) | 0.001 |
| TP53 status | |  |  |  |  |  |  |  |  |  |  |  |  |
|  | mutated | 0.5 (0.22 − 1.11) | 0.083 | 0.51 (0.24 − 1.09) | 0.079 | 1.63 (0.65 − 4.08) | 0.290 | 0.5 (0.2 − 1.24) | 0.130 | 1.63 (0.75 − 3.52) | 0.210 | 1.81 (0.85 − 3.86) | 0.120 |
|  | wild type | 2.76 (1.44 − 5.31) | 0.001 | 8.45 (2.03 − 35.13) | 0.000 | 3.82 (1.88 − 7.74) | <0.0001 | 2.52 (1.32 − 4.81) | 0.004 | 11.17 (2.68 − 46.49) | <0.0001 | 3.23 (1.69 − 6.17) | 0.000 |
| Endocrine therapy | |  |  |  |  |  |  |  |  |  |  |  |  |
|  | Tamoxifen only | 2.06 (1.01 − 4.18) | 0.042 | 1.58 (0.73 − 3.42) | 0.240 | 3.47 (1.33 − 9.06) | 0.007 | 2.42 (1.16 − 5.04) | 0.015 | 3.91 (1.19 − 12.83) | 0.015 | 3.07 (1.37 − 6.88) | 0.004 |
|  | any endocrine therapy | 1.68 (0.98 − 2.89) | 0.058 | 1.55 (0.87 − 2.77) | 0.130 | 2.51 (1.22 − 5.15) | 0.010 | 2.54 (1.42 − 4.53) | 0.001 | 3.55 (1.41 − 8.93) | 0.004 | 2.89 (1.41 − 5.94) | 0.002 |
|  | no endocrine therapy | 2.84 (1.89 − 4.27) | <0.0001 | 2.29 (1.52 − 3.45) | <0.0001 | 2.75 (1.88 − 4.03) | <0.0001 | 2.47 (1.58 − 3.84) | <0.0001 | 3.55 (2.23 − 5.67) | <0.0001 | 2.84 (1.88 − 4.28) | <0.0001 |
| Chemotherapy | |  |  |  |  |  |  |  |  |  |  |  |  |
|  | any chemotherapy | 1.81 (1.06 − 3.08) | 0.027 | 0.81 (0.47 − 1.4) | 0.450 | 1.72 (0.98 − 3.05) | 0.058 | 0.69 (0.38 − 1.27) | 0.230 | 1.5 (0.83 − 2.7) | 0.180 | 1.67 (0.99 − 2.82) | 0.050 |
|  | adjuvant only | 2.42 (1.33 − 4.43) | 0.003 | 0.78 (0.4 − 1.51) | 0.450 | 1.77 (0.94 − 3.35) | 0.073 | 0.6 (0.28 − 1.3) | 0.190 | 1.3 (0.68 − 2.48) | 0.430 | 1.96 (1.02 − 3.75) | 0.038 |
|  | neoadjuvant only | 0.61 (0.27 − 1.39) | 0.230 | 0.54 (0.25 − 1.15) | 0.100 | 0.58 (0.24 − 1.38) | 0.210 | 1.58 (0.74 − 3.37) | 0.230 | 0.59 (0.27 − 1.3) | 0.190 | 2.11 (0.98 − 4.54) | 0.052 |
|  | no chemotherapy | 2.13 (1.49 − 3.04) | <0.0001 | 1.9 (1.28 − 2.83) | 0.001 | 2.71 (1.83 − 4.02) | <0.0001 | 2.61 (1.76 − 3.87) | <0.0001 | 3.86 (2.37 − 6.29) | <0.0001 | 2.85 (1.93 − 4.22) | <0.0001 |
|  |  |  |  |  |  |  |  |  |  |  |  |  |  |
| **DMFS** | | 2.02 (1.66 − 2.46) | <0.0001 | 1.72 (1.42 − 2.09) | <0.0001 | 2.19 (1.72 − 2.8) | <0.0001 | 2.27 (1.8 − 2.85) | <0.0001 | 1.99 (1.64 − 2.42) | <0.0001 | 2 (1.62 − 2.46) | <0.0001 |
| ER status | |  |  |  |  |  |  |  |  |  |  |  |  |
|  | ER positive | 2.33 (1.66 − 3.29) | <0.0001 | 2.72 (1.93 − 3.82) | <0.0001 | 2.68 (1.9 − 3.77) | <0.0001 | 4.08 (2.74 − 6.09) | <0.0001 | 3.37 (2.4 − 4.74) | <0.0001 | 2.9 (2.05 − 4.09) | <0.0001 |
|  | ER negative | 0.72 (0.4 − 1.3) | 0.270 | 0.61 (0.38 − 0.98) | 0.041 | 0.58 (0.34 − 0.99) | 0.041 | 0.72 (0.4 − 1.29) | 0.270 | 0.56 (0.35 − 0.88) | 0.011 | 0.48 (0.29 − 0.79) | 0.003 |
| PR status | |  |  |  |  |  |  |  |  |  |  |  |  |
|  | PR positive | 1.89 (0.78 − 4.61) | 0.150 | 4.06 (1.37 − 12.05) | 0.006 | 5.08 (1.19 − 21.74) | 0.015 | 7.47 (1.75 − 31.88) | 0.001 | 9.6 (1.29 − 71.24) | 0.007 | 4.98 (1.48 − 16.8) | 0.004 |
|  | PR negative | 2.32 (1.07 − 5.03) | 0.028 | 1.95 (0.9 − 4.21) | 0.086 | 0.67 (0.32 − 1.39) | 0.280 | 2.03 (0.98 − 4.21) | 0.052 | 2.15 (1 − 4.62) | 0.045 | 2.42 (1.08 − 5.45) | 0.027 |
| HER2 status | |  |  |  |  |  |  |  |  |  |  |  |  |
|  | HER2 positive | 1.8 (0.95 − 3.41) | 0.068 | 1.61 (0.85 − 3.07) | 0.140 | 1.38 (0.7 − 2.69) | 0.350 | 1.83 (0.84 − 3.99) | 0.120 | 1.87 (0.88 − 3.95) | 0.096 | 1.67 (0.73 − 3.79) | 0.220 |
|  | HER2 negative | 3.07 (1.29 − 7.28) | 0.008 | 4.1 (1.48 − 11.36) | 0.003 | 1.85 (0.76 − 4.47) | 0.170 | 2.7 (1.14 − 6.38) | 0.019 | 2.88 (0.96 − 8.59) | 0.048 | 4.12 (1.2 − 14.12) | 0.015 |
| Lymph node status | |  |  |  |  |  |  |  |  |  |  |  |  |
|  | Lymph node positive | 2.32 (1.56 − 3.45) | <0.0001 | 2.13 (1.44 − 3.15) | <0.0001 | 1.82 (1.2 − 2.77) | 0.004 | 2.14 (1.37 − 3.34) | 0.001 | 1.98 (1.34 − 2.92) | 0.001 | 3.04 (1.82 − 5.07) | <0.0001 |
|  | Lymph node negative | 2.07 (1.56 − 2.75) | <0.0001 | 1.91 (1.45 − 2.51) | <0.0001 | 2.38 (1.74 − 3.25) | <0.0001 | 2.32 (1.73 − 3.13) | <0.0001 | 2.29 (1.72 − 3.04) | <0.0001 | 1.86 (1.42 − 2.45) | <0.0001 |
| Grade | |  |  |  |  |  |  |  |  |  |  |  |  |
|  | I | 2.83 (1.24 − 6.48) | 0.010 | 2.74 (1.01 − 7.45) | 0.039 | 5.01 (2.04 − 12.3) | <0.0001 | 3.98 (1.69 − 9.35) | 0.001 | 7.17 (2.99 − 17.2) | <0.0001 | 4.33 (1.85 − 10.16) | 0.000 |
|  | II | 2.23 (1.57 − 3.17) | <0.0001 | 2.27 (1.59 − 3.24) | <0.0001 | 2.05 (1.44 − 2.91) | <0.0001 | 2.49 (1.73 − 3.6) | <0.0001 | 2.19 (1.53 − 3.14) | <0.0001 | 2.12 (1.49 − 3.02) | <0.0001 |
|  | III | 1.42 (0.99 − 2.03) | 0.054 | 1.4 (0.95 − 2.05) | 0.084 | 1.33 (0.88 − 2.01) | 0.170 | 1.39 (0.98 − 1.97) | 0.066 | 1.31 (0.9 − 1.9) | 0.150 | 1.38 (0.91 − 2.08) | 0.130 |
| Intrinsic subtype | |  |  |  |  |  |  |  |  |  |  |  |  |
|  | Luminal A | 2.22 (1.56 − 3.15) | <0.0001 | 1.75 (1.3 − 2.37) | 0.000 | 2.4 (1.75 − 3.28) | <0.0001 | 2.55 (1.88 − 3.45) | <0.0001 | 2.21 (1.66 − 2.95) | <0.0001 | 2.11 (1.57 − 2.83) | <0.0001 |
|  | Luminal B | 1.7 (1.18 − 2.44) | 0.004 | 1.56 (1.08 − 2.26) | 0.018 | 1.57 (1.1 − 2.23) | 0.012 | 1.58 (1.09 − 2.29) | 0.015 | 1.89 (1.33 − 2.68) | 0.000 | 1.39 (0.91 − 2.13) | 0.130 |
|  | HER-2 | 1.68 (0.87 − 3.27) | 0.120 | 1.81 (0.95 − 3.44) | 0.065 | 0.52 (0.28 − 0.99) | 0.042 | 1.85 (0.96 − 3.54) | 0.060 | 1.5 (0.8 − 2.79) | 0.200 | 1.36 (0.71 − 2.6) | 0.350 |
|  | TNBC | 0.83 (0.5 − 1.39) | 0.480 | 0.55 (0.33 − 0.92) | 0.020 | 0.48 (0.28 − 0.79) | 0.004 | 0.52 (0.32 − 0.87) | 0.011 | 0.54 (0.32 − 0.89) | 0.015 | 0.5 (0.3 − 0.83) | 0.006 |
| TP53 status | |  |  |  |  |  |  |  |  |  |  |  |  |
|  | mutated | 2.76 (0.8 − 9.45) | 0.093 | 0.33 (0.12 − 0.9) | 0.023 | 0.64 (0.23 − 1.78) | 0.390 | 0.46 (0.19 − 1.14) | 0.085 | 0.64 (0.25 − 1.67) | 0.360 | 0.67 (0.27 − 1.69) | 0.400 |
|  | wild type | 4.64 (1.75 − 12.35) | 0.001 | 3.81 (1.53 − 9.5) | 0.002 | 4.25 (1.6 − 11.3) | 0.002 | 4.62 (2.05 − 10.4) | <0.0001 | 8.04 (1.9 − 34.05) | 0.001 | 2.98 (1.35 − 6.6) | 0.005 |
| Endocrine therapy | |  |  |  |  |  |  |  |  |  |  |  |  |
|  | Tamoxifen only | 2.02 (1.42 − 2.89) | <0.0001 | 2.27 (1.59 − 3.24) | <0.0001 | 2.49 (1.73 − 3.6) | <0.0001 | 3.28 (2.08 − 5.15) | <0.0001 | 2.81 (1.98 − 3.98) | <0.0001 | 2.46 (1.73 − 3.5) | <0.0001 |
|  | any endocrine therapy | 2.13 (1.51 − 3.02) | <0.0001 | 2.38 (1.68 − 3.37) | <0.0001 | 2.51 (1.75 − 3.61) | <0.0001 | 3.38 (2.15 − 5.3) | <0.0001 | 2.87 (2.03 − 4.05) | <0.0001 | 2.44 (1.73 − 3.44) | <0.0001 |
|  | no endocrine therapy | 2.42 (1.76 − 3.31) | <0.0001 | 1.64 (1.24 − 2.17) | 0.000 | 1.88 (1.42 − 2.5) | <0.0001 | 1.99 (1.49 − 2.67) | <0.0001 | 1.92 (1.42 − 2.6) | <0.0001 | 2.07 (1.52 − 2.83) | <0.0001 |
| Chemotherapy | |  |  |  |  |  |  |  |  |  |  |  |  |
|  | any chemotherapy | 1.99 (1.08 − 3.63) | 0.023 | 1.5 (0.92 − 2.45) | 0.100 | 0.6 (0.33 − 1.1) | 0.096 | 1.28 (0.8 − 2.05) | 0.300 | 1.36 (0.84 − 2.22) | 0.210 | 1.4 (0.8 − 2.45) | 0.240 |
|  | adjuvant only | 2.52 (1.42 − 4.47) | 0.001 | 1.79 (0.96 − 3.35) | 0.064 | 1.4 (0.76 − 2.56) | 0.280 | 2.1 (1.01 − 4.35) | 0.042 | 1.63 (0.91 − 2.94) | 0.099 | 1.78 (0.92 − 3.43) | 0.082 |
|  | neoadjuvant only | 0.55 (0.25 − 1.24) | 0.140 | 0.45 (0.2 − 1) | 0.045 | 0.37 (0.13 − 1.08) | 0.058 | 0.31 (0.09 − 1.04) | 0.046 | 0.59 (0.26 − 1.31) | 0.190 | 0.65 (0.29 − 1.45) | 0.290 |
|  | no chemotherapy | 2.04 (1.58 − 2.65) | <0.0001 | 1.99 (1.54 − 2.56) | <0.0001 | 2.29 (1.77 − 2.95) | <0.0001 | 2.61 (1.97 − 3.45) | <0.0001 | 2.41 (1.85 − 3.15) | <0.0001 | 2.1 (1.64 − 2.68) | <0.0001 |
