# Supplementary material for: Overexpression of kinesin superfamily members as prognostic biomarkers of breast cancer
Source: Cancer Cell Int. 2020 Apr 15;20:123. doi: 10.1186/s12935-020-01191-1 (PMC7161125; doi:10.1186/s12935-020-01191-1)
Supplement: Supplementary file 6 — Additional file 6. Clinical characters of patients enrolled. [file 12935_2020_1191_MOESM6_ESM.docx]

**Additional file 6. Patients characteristics.**

|  | | Patient Number | |
| --- | --- | --- | --- |
|  |  | n | % |
| Gender | | | |
|  | Female | 30 | 100% |
| Age | | | |
|  | <45 | 8 | 27% |
|  | ≥45; <60 | 9 | 30% |
|  | ≥60 | 13 | 43% |
| T | | | |
|  | T1 | 14 | 47% |
|  | T2 | 14 | 47% |
|  | T3 | 2 | 6% |
| N | | | |
|  | N0 | 16 | 53% |
|  | N1 | 8 | 27% |
|  | N2 | 4 | 13% |
|  | N3 | 2 | 7% |
| M | | | |
|  | M0 | 30 | 100% |
| Stage | | | |
|  | IA | 9 | 30% |
|  | IB | 1 | 3% |
|  | IIA | 9 | 30% |
|  | IIB | 5 | 17% |
|  | IIIA | 4 | 13% |
|  | IIIC | 2 | 7% |
| Nuclear Grade | | | |
|  | II | 14 | 47% |
|  | III | 16 | 53% |
| ER | | | |
|  | Positive | 20 | 67% |
|  | Negative | 10 | 33% |
| PR | | | |
|  | Positive | 21 | 70% |
|  | Negative | 9 | 30% |
| HER2 | | | |
|  | Positive | 10 | 33% |
|  | Negative | 20 | 67% |
| Ki-67 | | | |
|  | <20% | 9 | 30% |
|  | ≥20%; <30% | 5 | 17% |
|  | ≥30% | 16 | 53% |
| Subtype | | | |
|  | Luminal A | 8 | 27% |
|  | Luminal B;HER2+ | 9 | 30% |
|  | Luminal B;HER2- | 6 | 20% |
|  | HER2 | 4 | 13% |
|  | Triple Negative | 3 | 10% |
