# Supplementary material for: Overexpression of kinesin superfamily members as prognostic biomarkers of breast cancer
Source: Cancer Cell Int. 2020 Apr 15;20:123. doi: 10.1186/s12935-020-01191-1 (PMC7161125; doi:10.1186/s12935-020-01191-1)
Supplement: Supplementary file 7 — Additional file 7. (1) GO enrichment results of the 6 KIFs selected by LASSO regression. (2) KEGG enrichment results of the 6 KIFs selected by LASSO regression. [file 12935_2020_1191_MOESM7_ESM.docx]

**Additional file 7-1: GO enrichment results of the 6 KIFs selected by LASSO regression.**

|  | ONTOLOGY | ID | Description | GeneRatio | BgRatio | pvalue | p.adjust | qvalue | geneID | Count |
| --- | --- | --- | --- | --- | --- | --- | --- | --- | --- | --- |
| GO:0000280 | BP | GO:0000280 | nuclear division | 25/83 | 416/18493 | 1.01E-21 | 1.16E-18 | 8.53E-19 | 3832/9493/11004/24137/3833/1062/9928/81930/146909/991/146956/9232/4605/898/1164/113130/9088/9212/79980/81620/11065/2175/84057/10460/9735 | 25 |
| GO:0048285 | BP | GO:0048285 | organelle fission | 25/83 | 458/18493 | 1.06E-20 | 6.03E-18 | 4.44E-18 | 3832/9493/11004/24137/3833/1062/9928/81930/146909/991/146956/9232/4605/898/1164/113130/9088/9212/79980/81620/11065/2175/84057/10460/9735 | 25 |
| GO:0140014 | BP | GO:0140014 | mitotic nuclear division | 21/83 | 277/18493 | 2.42E-20 | 9.21E-18 | 6.78E-18 | 3832/9493/11004/24137/3833/1062/9928/81930/146909/991/9232/4605/898/113130/9088/9212/79980/81620/11065/10460/9735 | 21 |
| GO:0007059 | BP | GO:0007059 | chromosome segregation | 21/83 | 312/18493 | 2.87E-19 | 8.20E-17 | 6.04E-17 | 9493/11004/24137/3833/1062/9928/81930/146909/2237/991/146956/9232/898/332/113130/9212/79980/81620/387103/10460/55839 | 21 |
| GO:0098813 | BP | GO:0098813 | nuclear chromosome segregation | 18/83 | 258/18493 | 7.36E-17 | 1.68E-14 | 1.24E-14 | 9493/11004/24137/3833/1062/9928/81930/146909/2237/991/146956/9232/898/113130/9212/79980/81620/10460 | 18 |
| GO:0006260 | BP | GO:0006260 | DNA replication | 18/83 | 268/18493 | 1.44E-16 | 2.46E-14 | 1.81E-14 | 2237/8208/146956/5984/23560/898/4172/4171/80119/5111/23594/81620/64785/84296/29980/1763/51514/144455 | 18 |
| GO:0000819 | BP | GO:0000819 | sister chromatid segregation | 16/83 | 185/18493 | 1.50E-16 | 2.46E-14 | 1.81E-14 | 9493/11004/24137/3833/1062/9928/81930/146909/2237/991/9232/113130/9212/79980/81620/10460 | 16 |
| GO:0000070 | BP | GO:0000070 | mitotic sister chromatid segregation | 15/83 | 153/18493 | 2.17E-16 | 3.11E-14 | 2.29E-14 | 9493/11004/24137/3833/1062/9928/81930/146909/991/9232/113130/9212/79980/81620/10460 | 15 |
| GO:0006261 | BP | GO:0006261 | DNA-dependent DNA replication | 14/83 | 148/18493 | 3.90E-15 | 4.95E-13 | 3.65E-13 | 2237/146956/5984/898/4172/4171/5111/23594/81620/64785/84296/29980/1763/144455 | 14 |
| GO:0007051 | BP | GO:0007051 | spindle organization | 14/83 | 165/18493 | 1.80E-14 | 2.06E-12 | 1.52E-12 | 3832/9493/24137/3833/1062/991/3925/4605/203068/284403/9212/10460/79000/6491 | 14 |
| GO:0007052 | BP | GO:0007052 | mitotic spindle organization | 12/83 | 108/18493 | 5.77E-14 | 5.99E-12 | 4.42E-12 | 3832/9493/24137/3833/1062/991/3925/4605/284403/9212/10460/6491 | 12 |
| GO:1902850 | BP | GO:1902850 | microtubule cytoskeleton organization involved in mitosis | 12/83 | 129/18493 | 5.02E-13 | 4.78E-11 | 3.52E-11 | 3832/9493/24137/3833/1062/991/3925/4605/284403/9212/10460/6491 | 12 |
| GO:0045787 | BP | GO:0045787 | positive regulation of cell cycle | 16/83 | 390/18493 | 1.61E-11 | 1.41E-09 | 1.04E-09 | 9493/9928/2237/1163/898/1164/5111/113130/9212/1869/81620/11065/10733/51514/144455/11113 | 16 |
| GO:0007088 | BP | GO:0007088 | regulation of mitotic nuclear division | 12/83 | 177/18493 | 2.18E-11 | 1.78E-09 | 1.31E-09 | 3832/1062/991/9232/898/113130/9088/9212/81620/11065/10460/9735 | 12 |
| GO:0006271 | BP | GO:0006271 | DNA strand elongation involved in DNA replication | 6/83 | 17/18493 | 8.09E-11 | 6.17E-09 | 4.54E-09 | 2237/5984/5111/64785/84296/1763 | 6 |
| GO:0051783 | BP | GO:0051783 | regulation of nuclear division | 12/83 | 201/18493 | 9.64E-11 | 6.89E-09 | 5.07E-09 | 3832/1062/991/9232/898/113130/9088/9212/81620/11065/10460/9735 | 12 |
| GO:1901990 | BP | GO:1901990 | regulation of mitotic cell cycle phase transition | 15/83 | 375/18493 | 1.06E-10 | 7.14E-09 | 5.26E-09 | 1062/9928/991/203068/5111/113130/9212/1869/81620/11065/29980/10733/9735/51514/144455 | 15 |
| GO:1901987 | BP | GO:1901987 | regulation of cell cycle phase transition | 15/83 | 412/18493 | 3.92E-10 | 2.49E-08 | 1.84E-08 | 1062/9928/991/203068/5111/113130/9212/1869/81620/11065/29980/10733/9735/51514/144455 | 15 |
| GO:0090068 | BP | GO:0090068 | positive regulation of cell cycle process | 13/83 | 286/18493 | 4.44E-10 | 2.67E-08 | 1.97E-08 | 9493/9928/2237/5111/113130/9212/1869/81620/11065/10733/51514/144455/11113 | 13 |
| GO:0051983 | BP | GO:0051983 | regulation of chromosome segregation | 9/83 | 103/18493 | 8.44E-10 | 4.67E-08 | 3.44E-08 | 11004/1062/2237/991/9232/113130/9212/81620/10460 | 9 |
| GO:0022616 | BP | GO:0022616 | DNA strand elongation | 6/83 | 24/18493 | 8.58E-10 | 4.67E-08 | 3.44E-08 | 2237/5984/5111/64785/84296/1763 | 6 |
| GO:0007080 | BP | GO:0007080 | mitotic metaphase plate congression | 7/83 | 44/18493 | 9.50E-10 | 4.93E-08 | 3.64E-08 | 11004/3833/1062/9928/81930/113130/81620 | 7 |
| GO:0033044 | BP | GO:0033044 | regulation of chromosome organization | 13/83 | 330/18493 | 2.53E-09 | 1.24E-07 | 9.13E-08 | 1062/2237/991/9232/1789/1736/4171/80119/113130/9212/22948/81620/10460 | 13 |
| GO:0033045 | BP | GO:0033045 | regulation of sister chromatid segregation | 8/83 | 80/18493 | 2.60E-09 | 1.24E-07 | 9.13E-08 | 1062/2237/991/9232/113130/9212/81620/10460 | 8 |
| GO:0000083 | BP | GO:0000083 | regulation of transcription involved in G1/S transition of mitotic cell cycle | 6/83 | 29/18493 | 2.98E-09 | 1.36E-07 | 1.00E-07 | 898/5111/7298/1869/81620/144455 | 6 |
| GO:0000075 | BP | GO:0000075 | cell cycle checkpoint | 11/83 | 216/18493 | 3.31E-09 | 1.45E-07 | 1.07E-07 | 991/146956/5111/9212/1869/81620/29980/9735/1763/51514/144455 | 11 |
| GO:0071103 | BP | GO:0071103 | DNA conformation change | 12/83 | 283/18493 | 4.82E-09 | 2.04E-07 | 1.50E-07 | 3148/8208/55723/4171/80119/113130/79682/387103/84296/55839/3070/1763 | 12 |
| GO:0051310 | BP | GO:0051310 | metaphase plate congression | 7/83 | 57/18493 | 6.25E-09 | 2.55E-07 | 1.88E-07 | 11004/3833/1062/9928/81930/113130/81620 | 7 |
| GO:0090307 | BP | GO:0090307 | mitotic spindle assembly | 7/83 | 60/18493 | 9.04E-09 | 3.56E-07 | 2.62E-07 | 3832/9493/24137/3833/991/4605/9212 | 7 |
| GO:0044786 | BP | GO:0044786 | cell cycle DNA replication | 7/83 | 62/18493 | 1.14E-08 | 4.35E-07 | 3.21E-07 | 2237/5984/5111/81620/29980/1763/144455 | 7 |
| GO:0051225 | BP | GO:0051225 | spindle assembly | 8/83 | 105/18493 | 2.29E-08 | 8.43E-07 | 6.21E-07 | 3832/9493/24137/3833/991/4605/203068/9212 | 8 |
| GO:0000082 | BP | GO:0000082 | G1/S transition of mitotic cell cycle | 11/83 | 263/18493 | 2.55E-08 | 9.12E-07 | 6.72E-07 | 9928/898/4172/4171/5111/7298/23594/1869/81620/1033/144455 | 11 |
| GO:0007018 | BP | GO:0007018 | microtubule-based movement | 11/83 | 269/18493 | 3.22E-08 | 1.11E-06 | 8.21E-07 | 3832/10112/9493/11004/24137/3833/1062/9928/56992/81930/146909 | 11 |
| GO:0000723 | BP | GO:0000723 | telomere maintenance | 9/83 | 157/18493 | 3.50E-08 | 1.18E-06 | 8.67E-07 | 2237/5984/898/1736/80119/5111/9212/22948/1763 | 9 |
| GO:0051303 | BP | GO:0051303 | establishment of chromosome localization | 7/83 | 74/18493 | 4.00E-08 | 1.31E-06 | 9.63E-07 | 11004/3833/1062/9928/81930/113130/81620 | 7 |
| GO:0050000 | BP | GO:0050000 | chromosome localization | 7/83 | 75/18493 | 4.40E-08 | 1.40E-06 | 1.03E-06 | 11004/3833/1062/9928/81930/113130/81620 | 7 |
| GO:0000910 | BP | GO:0000910 | cytokinesis | 9/83 | 163/18493 | 4.84E-08 | 1.50E-06 | 1.10E-06 | 10112/9493/24137/9928/3925/9212/81620/144455/11113 | 9 |
| GO:0044843 | BP | GO:0044843 | cell cycle G1/S phase transition | 11/83 | 282/18493 | 5.21E-08 | 1.57E-06 | 1.15E-06 | 9928/898/4172/4171/5111/7298/23594/1869/81620/1033/144455 | 11 |
| GO:0007093 | BP | GO:0007093 | mitotic cell cycle checkpoint | 9/83 | 166/18493 | 5.67E-08 | 1.66E-06 | 1.22E-06 | 991/146956/5111/9212/1869/81620/29980/9735/144455 | 9 |
| GO:0032200 | BP | GO:0032200 | telomere organization | 9/83 | 170/18493 | 6.96E-08 | 1.99E-06 | 1.46E-06 | 2237/5984/898/1736/80119/5111/9212/22948/1763 | 9 |
| GO:0033260 | BP | GO:0033260 | nuclear DNA replication | 6/83 | 51/18493 | 1.04E-07 | 2.91E-06 | 2.14E-06 | 2237/5984/5111/81620/29980/1763 | 6 |
| GO:0006890 | BP | GO:0006890 | retrograde vesicle-mediated transport Golgi to ER | 7/83 | 86/18493 | 1.14E-07 | 3.12E-06 | 2.30E-06 | 3832/9493/11004/24137/1062/56992/81930 | 7 |
| GO:0051304 | BP | GO:0051304 | chromosome separation | 7/83 | 89/18493 | 1.45E-07 | 3.86E-06 | 2.84E-06 | 1062/991/146956/9232/9212/81620/10460 | 7 |
| GO:0065004 | BP | GO:0065004 | protein-DNA complex assembly | 10/83 | 254/18493 | 2.02E-07 | 5.22E-06 | 3.85E-06 | 1062/3148/8208/55723/4171/81620/79682/387103/55839/3070 | 10 |
| GO:0010965 | BP | GO:0010965 | regulation of mitotic sister chromatid separation | 6/83 | 57/18493 | 2.06E-07 | 5.22E-06 | 3.85E-06 | 1062/991/9232/9212/81620/10460 | 6 |
| GO:0051306 | BP | GO:0051306 | mitotic sister chromatid separation | 6/83 | 60/18493 | 2.81E-07 | 6.72E-06 | 4.95E-06 | 1062/991/9232/9212/81620/10460 | 6 |
| GO:0019886 | BP | GO:0019886 | antigen processing and presentation of exogenous peptide antigen via MHC class II | 7/83 | 98/18493 | 2.82E-07 | 6.72E-06 | 4.95E-06 | 3832/9493/11004/24137/1062/56992/81930 | 7 |
| GO:0061640 | BP | GO:0061640 | cytoskeleton-dependent cytokinesis | 7/83 | 98/18493 | 2.82E-07 | 6.72E-06 | 4.95E-06 | 10112/9493/24137/3925/9212/81620/11113 | 7 |
| GO:0006270 | BP | GO:0006270 | DNA replication initiation | 5/83 | 32/18493 | 2.95E-07 | 6.77E-06 | 4.99E-06 | 898/4172/4171/23594/81620 | 5 |
| GO:0051052 | BP | GO:0051052 | regulation of DNA metabolic process | 12/83 | 412/18493 | 2.96E-07 | 6.77E-06 | 4.99E-06 | 5984/23560/1736/80119/5111/9212/22948/81620/29980/79000/1763/144455 | 12 |
| GO:1905818 | BP | GO:1905818 | regulation of chromosome separation | 6/83 | 62/18493 | 3.42E-07 | 7.63E-06 | 5.62E-06 | 1062/991/9232/9212/81620/10460 | 6 |
| GO:0002495 | BP | GO:0002495 | antigen processing and presentation of peptide antigen via MHC class II | 7/83 | 101/18493 | 3.47E-07 | 7.63E-06 | 5.62E-06 | 3832/9493/11004/24137/1062/56992/81930 | 7 |
| GO:0002504 | BP | GO:0002504 | antigen processing and presentation of peptide or polysaccharide antigen via MHC class II | 7/83 | 102/18493 | 3.71E-07 | 8.01E-06 | 5.90E-06 | 3832/9493/11004/24137/1062/56992/81930 | 7 |
| GO:0006323 | BP | GO:0006323 | DNA packaging | 9/83 | 209/18493 | 4.04E-07 | 8.55E-06 | 6.30E-06 | 3148/8208/55723/4171/113130/79682/387103/55839/3070 | 9 |
| GO:0045930 | BP | GO:0045930 | negative regulation of mitotic cell cycle | 10/83 | 281/18493 | 5.12E-07 | 1.05E-05 | 7.75E-06 | 991/146956/9232/5111/9212/1869/81620/29980/9735/144455 | 10 |
| GO:0006275 | BP | GO:0006275 | regulation of DNA replication | 7/83 | 107/18493 | 5.15E-07 | 1.05E-05 | 7.75E-06 | 5984/23560/5111/81620/29980/1763/144455 | 7 |
| GO:0031570 | BP | GO:0031570 | DNA integrity checkpoint | 8/83 | 158/18493 | 5.45E-07 | 1.09E-05 | 8.05E-06 | 146956/5111/1869/81620/29980/1763/51514/144455 | 8 |
| GO:0033047 | BP | GO:0033047 | regulation of mitotic sister chromatid segregation | 6/83 | 68/18493 | 5.96E-07 | 1.18E-05 | 8.66E-06 | 1062/991/9232/9212/81620/10460 | 6 |
| GO:0051302 | BP | GO:0051302 | regulation of cell division | 8/83 | 162/18493 | 6.59E-07 | 1.28E-05 | 9.40E-06 | 10112/9493/9928/146909/9212/23594/144455/11113 | 8 |
| GO:0071824 | BP | GO:0071824 | protein-DNA complex subunit organization | 10/83 | 290/18493 | 6.83E-07 | 1.30E-05 | 9.58E-06 | 1062/3148/8208/55723/4171/81620/79682/387103/55839/3070 | 10 |
| GO:0031497 | BP | GO:0031497 | chromatin assembly | 8/83 | 166/18493 | 7.93E-07 | 1.49E-05 | 1.09E-05 | 3148/8208/55723/4171/79682/387103/55839/3070 | 8 |
| GO:0140013 | BP | GO:0140013 | meiotic nuclear division | 8/83 | 171/18493 | 9.92E-07 | 1.83E-05 | 1.35E-05 | 81930/991/146956/9232/898/1164/2175/84057 | 8 |
| GO:0002478 | BP | GO:0002478 | antigen processing and presentation of exogenous peptide antigen | 7/83 | 126/18493 | 1.56E-06 | 2.83E-05 | 2.08E-05 | 3832/9493/11004/24137/1062/56992/81930 | 7 |
| GO:0051321 | BP | GO:0051321 | meiotic cell cycle | 9/83 | 249/18493 | 1.74E-06 | 3.10E-05 | 2.29E-05 | 81930/991/146956/9232/898/1164/9088/2175/84057 | 9 |
| GO:1903046 | BP | GO:1903046 | meiotic cell cycle process | 8/83 | 188/18493 | 2.02E-06 | 3.56E-05 | 2.62E-05 | 81930/991/146956/9232/898/1164/2175/84057 | 8 |
| GO:0032465 | BP | GO:0032465 | regulation of cytokinesis | 6/83 | 84/18493 | 2.09E-06 | 3.58E-05 | 2.64E-05 | 10112/9493/9928/9212/144455/11113 | 6 |
| GO:0071897 | BP | GO:0071897 | DNA biosynthetic process | 8/83 | 189/18493 | 2.10E-06 | 3.58E-05 | 2.64E-05 | 5984/1736/80119/5111/7298/9212/22948/51514 | 8 |
| GO:0051383 | BP | GO:0051383 | kinetochore organization | 4/83 | 21/18493 | 2.13E-06 | 3.58E-05 | 2.64E-05 | 1062/81620/387103/55839 | 4 |
| GO:0000281 | BP | GO:0000281 | mitotic cytokinesis | 6/83 | 85/18493 | 2.24E-06 | 3.72E-05 | 2.74E-05 | 10112/9493/24137/3925/81620/11113 | 6 |
| GO:0019884 | BP | GO:0019884 | antigen processing and presentation of exogenous antigen | 7/83 | 134/18493 | 2.35E-06 | 3.81E-05 | 2.81E-05 | 3832/9493/11004/24137/1062/56992/81930 | 7 |
| GO:0006333 | BP | GO:0006333 | chromatin assembly or disassembly | 8/83 | 192/18493 | 2.37E-06 | 3.81E-05 | 2.81E-05 | 3148/8208/55723/4171/79682/387103/55839/3070 | 8 |
| GO:0000079 | BP | GO:0000079 | regulation of cyclin-dependent protein serine/threonine kinase activity | 6/83 | 87/18493 | 2.57E-06 | 4.08E-05 | 3.01E-05 | 1163/23560/898/1164/9088/1033 | 6 |
| GO:0048002 | BP | GO:0048002 | antigen processing and presentation of peptide antigen | 7/83 | 139/18493 | 3.00E-06 | 4.70E-05 | 3.47E-05 | 3832/9493/11004/24137/1062/56992/81930 | 7 |
| GO:0000086 | BP | GO:0000086 | G2/M transition of mitotic cell cycle | 8/83 | 199/18493 | 3.09E-06 | 4.77E-05 | 3.51E-05 | 9928/203068/9088/9212/29980/10733/51514/11113 | 8 |
| GO:1904029 | BP | GO:1904029 | regulation of cyclin-dependent protein kinase activity | 6/83 | 91/18493 | 3.35E-06 | 5.10E-05 | 3.76E-05 | 1163/23560/898/1164/9088/1033 | 6 |
| GO:0032201 | BP | GO:0032201 | telomere maintenance via semi-conservative replication | 4/83 | 24/18493 | 3.74E-06 | 5.63E-05 | 4.15E-05 | 2237/5984/5111/1763 | 4 |
| GO:2001251 | BP | GO:2001251 | negative regulation of chromosome organization | 7/83 | 144/18493 | 3.80E-06 | 5.64E-05 | 4.15E-05 | 991/9232/1789/4171/80119/9212/81620 | 7 |
| GO:0006334 | BP | GO:0006334 | nucleosome assembly | 7/83 | 146/18493 | 4.16E-06 | 6.10E-05 | 4.49E-05 | 3148/8208/55723/4171/79682/387103/55839 | 7 |
| GO:0007091 | BP | GO:0007091 | metaphase/anaphase transition of mitotic cell cycle | 5/83 | 54/18493 | 4.29E-06 | 6.21E-05 | 4.57E-05 | 1062/991/9212/81620/10460 | 5 |
| GO:1901988 | BP | GO:1901988 | negative regulation of cell cycle phase transition | 8/83 | 211/18493 | 4.76E-06 | 6.81E-05 | 5.01E-05 | 991/5111/9212/1869/81620/29980/51514/144455 | 8 |
| GO:0034508 | BP | GO:0034508 | centromere complex assembly | 5/83 | 56/18493 | 5.15E-06 | 7.17E-05 | 5.28E-05 | 1062/79682/387103/55839/3070 | 5 |
| GO:0044784 | BP | GO:0044784 | metaphase/anaphase transition of cell cycle | 5/83 | 56/18493 | 5.15E-06 | 7.17E-05 | 5.28E-05 | 1062/991/9212/81620/10460 | 5 |
| GO:0044839 | BP | GO:0044839 | cell cycle G2/M phase transition | 8/83 | 218/18493 | 6.06E-06 | 8.34E-05 | 6.15E-05 | 9928/203068/9088/9212/29980/10733/51514/11113 | 8 |
| GO:0051054 | BP | GO:0051054 | positive regulation of DNA metabolic process | 8/83 | 220/18493 | 6.48E-06 | 8.81E-05 | 6.49E-05 | 5984/1736/5111/9212/22948/81620/1763/144455 | 8 |
| GO:0010948 | BP | GO:0010948 | negative regulation of cell cycle process | 9/83 | 304/18493 | 8.82E-06 | 0.000118578 | 8.74E-05 | 991/9232/5111/9212/1869/81620/29980/51514/144455 | 9 |
| GO:0051256 | BP | GO:0051256 | mitotic spindle midzone assembly | 3/83 | 10/18493 | 1.02E-05 | 0.000135918 | 0.000100138 | 9493/24137/9212 | 3 |
| GO:0008608 | BP | GO:0008608 | attachment of spindle microtubules to kinetochore | 4/83 | 32/18493 | 1.23E-05 | 0.000161936 | 0.000119306 | 11004/1062/9212/81620 | 4 |
| GO:0019882 | BP | GO:0019882 | antigen processing and presentation | 7/83 | 175/18493 | 1.37E-05 | 0.000177448 | 0.000130734 | 3832/9493/11004/24137/1062/56992/81930 | 7 |
| GO:0034728 | BP | GO:0034728 | nucleosome organization | 7/83 | 181/18493 | 1.70E-05 | 0.000218297 | 0.00016083 | 3148/8208/55723/4171/79682/387103/55839 | 7 |
| GO:0000022 | BP | GO:0000022 | mitotic spindle elongation | 3/83 | 12/18493 | 1.86E-05 | 0.000236568 | 0.000174292 | 9493/24137/9212 | 3 |
| GO:0031145 | BP | GO:0031145 | anaphase-promoting complex-dependent catabolic process | 4/83 | 37/18493 | 2.23E-05 | 0.000273518 | 0.000201515 | 991/9232/9212/11065 | 4 |
| GO:0032467 | BP | GO:0032467 | positive regulation of cytokinesis | 4/83 | 37/18493 | 2.23E-05 | 0.000273518 | 0.000201515 | 9493/9928/9212/11113 | 4 |
| GO:0045740 | BP | GO:0045740 | positive regulation of DNA replication | 4/83 | 37/18493 | 2.23E-05 | 0.000273518 | 0.000201515 | 5111/81620/1763/144455 | 4 |
| GO:0032392 | BP | GO:0032392 | DNA geometric change | 5/83 | 76/18493 | 2.32E-05 | 0.000281518 | 0.000207409 | 3148/4171/80119/84296/1763 | 5 |
| GO:1901991 | BP | GO:1901991 | negative regulation of mitotic cell cycle phase transition | 7/83 | 191/18493 | 2.40E-05 | 0.000281518 | 0.000207409 | 991/5111/9212/1869/81620/29980/144455 | 7 |
| GO:0051231 | BP | GO:0051231 | spindle elongation | 3/83 | 13/18493 | 2.41E-05 | 0.000281518 | 0.000207409 | 9493/24137/9212 | 3 |
| GO:0051255 | BP | GO:0051255 | spindle midzone assembly | 3/83 | 13/18493 | 2.41E-05 | 0.000281518 | 0.000207409 | 9493/24137/9212 | 3 |
| GO:0051315 | BP | GO:0051315 | attachment of mitotic spindle microtubules to kinetochore | 3/83 | 13/18493 | 2.41E-05 | 0.000281518 | 0.000207409 | 11004/1062/81620 | 3 |
| GO:2000816 | BP | GO:2000816 | negative regulation of mitotic sister chromatid separation | 4/83 | 39/18493 | 2.75E-05 | 0.000317813 | 0.000234149 | 991/9232/9212/81620 | 4 |
| GO:1905819 | BP | GO:1905819 | negative regulation of chromosome separation | 4/83 | 40/18493 | 3.05E-05 | 0.000348403 | 0.000256686 | 991/9232/9212/81620 | 4 |
| GO:0034502 | BP | GO:0034502 | protein localization to chromosome | 5/83 | 81/18493 | 3.16E-05 | 0.000357786 | 0.000263599 | 1736/113130/9212/22948/81620 | 5 |
| GO:0007019 | BP | GO:0007019 | microtubule depolymerization | 4/83 | 41/18493 | 3.37E-05 | 0.000377209 | 0.000277909 | 11004/81930/146909/3925 | 4 |
| GO:0033048 | BP | GO:0033048 | negative regulation of mitotic sister chromatid segregation | 4/83 | 42/18493 | 3.71E-05 | 0.000407505 | 0.00030023 | 991/9232/9212/81620 | 4 |
| GO:0045005 | BP | GO:0045005 | DNA-dependent DNA replication maintenance of fidelity | 4/83 | 42/18493 | 3.71E-05 | 0.000407505 | 0.00030023 | 146956/5111/29980/1763 | 4 |
| GO:0006310 | BP | GO:0006310 | DNA recombination | 8/83 | 282/18493 | 3.88E-05 | 0.000422629 | 0.000311372 | 2237/3148/146956/86/80119/84296/84057/79000 | 8 |
| GO:0033046 | BP | GO:0033046 | negative regulation of sister chromatid segregation | 4/83 | 44/18493 | 4.47E-05 | 0.000481609 | 0.000354826 | 991/9232/9212/81620 | 4 |
| GO:0051382 | BP | GO:0051382 | kinetochore assembly | 3/83 | 16/18493 | 4.68E-05 | 0.000499971 | 0.000368353 | 1062/387103/55839 | 3 |
| GO:0051985 | BP | GO:0051985 | negative regulation of chromosome segregation | 4/83 | 45/18493 | 4.89E-05 | 0.00051704 | 0.000380929 | 991/9232/9212/81620 | 4 |
| GO:0000077 | BP | GO:0000077 | DNA damage checkpoint | 6/83 | 146/18493 | 5.00E-05 | 0.000524466 | 0.0003864 | 146956/5111/1869/29980/51514/144455 | 6 |
| GO:0051782 | BP | GO:0051782 | negative regulation of cell division | 3/83 | 17/18493 | 5.66E-05 | 0.000588638 | 0.000433679 | 9212/23594/144455 | 3 |
| GO:0031055 | BP | GO:0031055 | chromatin remodeling at centromere | 4/83 | 48/18493 | 6.32E-05 | 0.000650293 | 0.000479104 | 79682/387103/55839/3070 | 4 |
| GO:0010389 | BP | GO:0010389 | regulation of G2/M transition of mitotic cell cycle | 6/83 | 153/18493 | 6.49E-05 | 0.000662563 | 0.000488143 | 9928/203068/9212/29980/10733/51514 | 6 |
| GO:0007096 | BP | GO:0007096 | regulation of exit from mitosis | 3/83 | 18/18493 | 6.78E-05 | 0.000685387 | 0.000504959 | 113130/11065/9735 | 3 |
| GO:0044773 | BP | GO:0044773 | mitotic DNA damage checkpoint | 5/83 | 98/18493 | 7.90E-05 | 0.000791585 | 0.0005832 | 146956/5111/1869/29980/144455 | 5 |
| GO:0030071 | BP | GO:0030071 | regulation of mitotic metaphase/anaphase transition | 4/83 | 51/18493 | 8.03E-05 | 0.000797924 | 0.000587871 | 1062/991/9212/81620 | 4 |
| GO:0045839 | BP | GO:0045839 | negative regulation of mitotic nuclear division | 4/83 | 52/18493 | 8.67E-05 | 0.000846751 | 0.000623844 | 991/9232/9212/81620 | 4 |
| GO:0090329 | BP | GO:0090329 | regulation of DNA-dependent DNA replication | 4/83 | 52/18493 | 8.67E-05 | 0.000846751 | 0.000623844 | 5984/81620/29980/144455 | 4 |
| GO:0032210 | BP | GO:0032210 | regulation of telomere maintenance via telomerase | 4/83 | 53/18493 | 9.34E-05 | 0.000897418 | 0.000661172 | 1736/80119/9212/22948 | 4 |
| GO:1902099 | BP | GO:1902099 | regulation of metaphase/anaphase transition of cell cycle | 4/83 | 53/18493 | 9.34E-05 | 0.000897418 | 0.000661172 | 1062/991/9212/81620 | 4 |
| GO:0006338 | BP | GO:0006338 | chromatin remodeling | 6/83 | 165/18493 | 9.86E-05 | 0.00093119 | 0.000686054 | 3148/86/79682/387103/55839/3070 | 6 |
| GO:2001252 | BP | GO:2001252 | positive regulation of chromosome organization | 6/83 | 165/18493 | 9.86E-05 | 0.00093119 | 0.000686054 | 2237/1789/1736/9212/22948/81620 | 6 |
| GO:0006336 | BP | GO:0006336 | DNA replication-independent nucleosome assembly | 4/83 | 54/18493 | 0.000100563 | 0.000942162 | 0.000694138 | 55723/79682/387103/55839 | 4 |
| GO:2000278 | BP | GO:2000278 | regulation of DNA biosynthetic process | 5/83 | 104/18493 | 0.000104689 | 0.000972843 | 0.000716742 | 5984/1736/80119/9212/22948 | 5 |
| GO:0034724 | BP | GO:0034724 | DNA replication-independent nucleosome organization | 4/83 | 55/18493 | 0.000108082 | 0.00099627 | 0.000734002 | 55723/79682/387103/55839 | 4 |
| GO:1902749 | BP | GO:1902749 | regulation of cell cycle G2/M phase transition | 6/83 | 170/18493 | 0.000116153 | 0.001062101 | 0.000782503 | 9928/203068/9212/29980/10733/51514 | 6 |
| GO:0044774 | BP | GO:0044774 | mitotic DNA integrity checkpoint | 5/83 | 107/18493 | 0.000119756 | 0.001081276 | 0.00079663 | 146956/5111/1869/29980/144455 | 5 |
| GO:0060249 | BP | GO:0060249 | anatomical structure homeostasis | 9/83 | 425/18493 | 0.000120142 | 0.001081276 | 0.00079663 | 2237/5984/898/1736/80119/5111/9212/22948/1763 | 9 |
| GO:0070507 | BP | GO:0070507 | regulation of microtubule cytoskeleton organization | 6/83 | 175/18493 | 0.000136132 | 0.001215613 | 0.000895603 | 3832/81930/3925/10460/10733/6491 | 6 |
| GO:0051784 | BP | GO:0051784 | negative regulation of nuclear division | 4/83 | 60/18493 | 0.000151924 | 0.001346121 | 0.000991754 | 991/9232/9212/81620 | 4 |
| GO:0007127 | BP | GO:0007127 | meiosis I | 5/83 | 113/18493 | 0.000154834 | 0.001361348 | 0.001002973 | 146956/9232/898/1164/84057 | 5 |
| GO:1904356 | BP | GO:1904356 | regulation of telomere maintenance via telomere lengthening | 4/83 | 61/18493 | 0.000162033 | 0.00141377 | 0.001041595 | 1736/80119/9212/22948 | 4 |
| GO:0061982 | BP | GO:0061982 | meiosis I cell cycle process | 5/83 | 118/18493 | 0.000189652 | 0.001642218 | 0.001209904 | 146956/9232/898/1164/84057 | 5 |
| GO:0032508 | BP | GO:0032508 | DNA duplex unwinding | 4/83 | 66/18493 | 0.000220004 | 0.001876599 | 0.001382584 | 4171/80119/84296/1763 | 4 |
| GO:2000573 | BP | GO:2000573 | positive regulation of DNA biosynthetic process | 4/83 | 66/18493 | 0.000220004 | 0.001876599 | 0.001382584 | 5984/1736/9212/22948 | 4 |
| GO:0007004 | BP | GO:0007004 | telomere maintenance via telomerase | 4/83 | 71/18493 | 0.000291595 | 0.002468837 | 0.001818915 | 1736/80119/9212/22948 | 4 |
| GO:0032886 | BP | GO:0032886 | regulation of microtubule-based process | 6/83 | 204/18493 | 0.000311991 | 0.002622098 | 0.001931831 | 3832/81930/3925/10460/10733/6491 | 6 |
| GO:0010458 | BP | GO:0010458 | exit from mitosis | 3/83 | 30/18493 | 0.000324284 | 0.002663155 | 0.001962079 | 113130/11065/9735 | 3 |
| GO:0072401 | BP | GO:0072401 | signal transduction involved in DNA integrity checkpoint | 4/83 | 73/18493 | 0.000324432 | 0.002663155 | 0.001962079 | 5111/1869/51514/144455 | 4 |
| GO:0072422 | BP | GO:0072422 | signal transduction involved in DNA damage checkpoint | 4/83 | 73/18493 | 0.000324432 | 0.002663155 | 0.001962079 | 5111/1869/51514/144455 | 4 |
| GO:0010639 | BP | GO:0010639 | negative regulation of organelle organization | 8/83 | 384/18493 | 0.000326196 | 0.002663155 | 0.001962079 | 991/9232/3925/1789/4171/80119/9212/81620 | 8 |
| GO:0072395 | BP | GO:0072395 | signal transduction involved in cell cycle checkpoint | 4/83 | 74/18493 | 0.000341807 | 0.002770818 | 0.0020414 | 5111/1869/51514/144455 | 4 |
| GO:0090305 | BP | GO:0090305 | nucleic acid phosphodiester bond hydrolysis | 7/83 | 295/18493 | 0.000360951 | 0.002905399 | 0.002140553 | 2237/10940/3148/146956/5984/5111/1763 | 7 |
| GO:0031297 | BP | GO:0031297 | replication fork processing | 3/83 | 32/18493 | 0.000393615 | 0.003146171 | 0.002317941 | 146956/5111/1763 | 3 |
| GO:0006278 | BP | GO:0006278 | RNA-dependent DNA biosynthetic process | 4/83 | 78/18493 | 0.000418014 | 0.003317988 | 0.002444528 | 1736/80119/9212/22948 | 4 |
| GO:0032204 | BP | GO:0032204 | regulation of telomere maintenance | 4/83 | 79/18493 | 0.000438815 | 0.00345907 | 0.00254847 | 1736/80119/9212/22948 | 4 |
| GO:0007094 | BP | GO:0007094 | mitotic spindle assembly checkpoint | 3/83 | 34/18493 | 0.000471817 | 0.003571436 | 0.002631256 | 991/9212/81620 | 3 |
| GO:0007099 | BP | GO:0007099 | centriole replication | 3/83 | 34/18493 | 0.000471817 | 0.003571436 | 0.002631256 | 284403/10733/6491 | 3 |
| GO:0031577 | BP | GO:0031577 | spindle checkpoint | 3/83 | 34/18493 | 0.000471817 | 0.003571436 | 0.002631256 | 991/9212/81620 | 3 |
| GO:0032212 | BP | GO:0032212 | positive regulation of telomere maintenance via telomerase | 3/83 | 34/18493 | 0.000471817 | 0.003571436 | 0.002631256 | 1736/9212/22948 | 3 |
| GO:0071173 | BP | GO:0071173 | spindle assembly checkpoint | 3/83 | 34/18493 | 0.000471817 | 0.003571436 | 0.002631256 | 991/9212/81620 | 3 |
| GO:0071174 | BP | GO:0071174 | mitotic spindle checkpoint | 3/83 | 34/18493 | 0.000471817 | 0.003571436 | 0.002631256 | 991/9212/81620 | 3 |
| GO:0043044 | BP | GO:0043044 | ATP-dependent chromatin remodeling | 4/83 | 81/18493 | 0.000482615 | 0.003629138 | 0.002673768 | 86/79682/387103/55839 | 4 |
| GO:0010833 | BP | GO:0010833 | telomere maintenance via telomere lengthening | 4/83 | 83/18493 | 0.000529445 | 0.003955265 | 0.002914042 | 1736/80119/9212/22948 | 4 |
| GO:0051781 | BP | GO:0051781 | positive regulation of cell division | 4/83 | 84/18493 | 0.000554032 | 0.004112072 | 0.003029569 | 9493/9928/9212/11113 | 4 |
| GO:0045841 | BP | GO:0045841 | negative regulation of mitotic metaphase/anaphase transition | 3/83 | 36/18493 | 0.000559336 | 0.004124652 | 0.003038838 | 991/9212/81620 | 3 |
| GO:1901992 | BP | GO:1901992 | positive regulation of mitotic cell cycle phase transition | 4/83 | 85/18493 | 0.000579421 | 0.004245374 | 0.003127779 | 113130/81620/11065/51514 | 4 |
| GO:0098534 | BP | GO:0098534 | centriole assembly | 3/83 | 37/18493 | 0.000606726 | 0.004361556 | 0.003213376 | 284403/10733/6491 | 3 |
| GO:1902100 | BP | GO:1902100 | negative regulation of metaphase/anaphase transition of cell cycle | 3/83 | 37/18493 | 0.000606726 | 0.004361556 | 0.003213376 | 991/9212/81620 | 3 |
| GO:1904358 | BP | GO:1904358 | positive regulation of telomere maintenance via telomere lengthening | 3/83 | 37/18493 | 0.000606726 | 0.004361556 | 0.003213376 | 1736/9212/22948 | 3 |
| GO:0032506 | BP | GO:0032506 | cytokinetic process | 3/83 | 38/18493 | 0.000656606 | 0.004661494 | 0.003434356 | 10112/9493/9212 | 3 |
| GO:0042769 | BP | GO:0042769 | DNA damage response detection of DNA damage | 3/83 | 38/18493 | 0.000656606 | 0.004661494 | 0.003434356 | 5984/5111/51514 | 3 |
| GO:0006302 | BP | GO:0006302 | double-strand break repair | 6/83 | 236/18493 | 0.000674374 | 0.00475808 | 0.003505516 | 2237/146956/113130/84296/79000/1763 | 6 |
| GO:0019985 | BP | GO:0019985 | translesion synthesis | 3/83 | 40/18493 | 0.000764047 | 0.005357701 | 0.003947286 | 5984/5111/51514 | 3 |
| GO:1903405 | BP | GO:1903405 | protein localization to nuclear body | 2/83 | 10/18493 | 0.000874911 | 0.006024234 | 0.004438354 | 1736/22948 | 2 |
| GO:1904851 | BP | GO:1904851 | positive regulation of establishment of protein localization to telomere | 2/83 | 10/18493 | 0.000874911 | 0.006024234 | 0.004438354 | 1736/22948 | 2 |
| GO:1904867 | BP | GO:1904867 | protein localization to Cajal body | 2/83 | 10/18493 | 0.000874911 | 0.006024234 | 0.004438354 | 1736/22948 | 2 |
| GO:0034080 | BP | GO:0034080 | CENP-A containing nucleosome assembly | 3/83 | 44/18493 | 0.001011054 | 0.006878779 | 0.005067941 | 79682/387103/55839 | 3 |
| GO:0061641 | BP | GO:0061641 | CENP-A containing chromatin organization | 3/83 | 44/18493 | 0.001011054 | 0.006878779 | 0.005067941 | 79682/387103/55839 | 3 |
| GO:0035404 | BP | GO:0035404 | histone-serine phosphorylation | 2/83 | 11/18493 | 0.001066221 | 0.007086847 | 0.005221235 | 7443/9212 | 2 |
| GO:0070203 | BP | GO:0070203 | regulation of establishment of protein localization to telomere | 2/83 | 11/18493 | 0.001066221 | 0.007086847 | 0.005221235 | 1736/22948 | 2 |
| GO:2000105 | BP | GO:2000105 | positive regulation of DNA-dependent DNA replication | 2/83 | 11/18493 | 0.001066221 | 0.007086847 | 0.005221235 | 81620/144455 | 2 |
| GO:1901989 | BP | GO:1901989 | positive regulation of cell cycle phase transition | 4/83 | 100/18493 | 0.001066437 | 0.007086847 | 0.005221235 | 113130/81620/11065/51514 | 4 |
| GO:0048193 | BP | GO:0048193 | Golgi vesicle transport | 7/83 | 359/18493 | 0.001149025 | 0.007591535 | 0.005593064 | 3832/9493/11004/24137/1062/56992/81930 | 7 |
| GO:0060707 | BP | GO:0060707 | trophoblast giant cell differentiation | 2/83 | 12/18493 | 0.001275739 | 0.008161998 | 0.006013352 | 10733/144455 | 2 |
| GO:0070202 | BP | GO:0070202 | regulation of establishment of protein localization to chromosome | 2/83 | 12/18493 | 0.001275739 | 0.008161998 | 0.006013352 | 1736/22948 | 2 |
| GO:1904668 | BP | GO:1904668 | positive regulation of ubiquitin protein ligase activity | 2/83 | 12/18493 | 0.001275739 | 0.008161998 | 0.006013352 | 991/11065 | 2 |
| GO:1904816 | BP | GO:1904816 | positive regulation of protein localization to chromosome telomeric region | 2/83 | 12/18493 | 0.001275739 | 0.008161998 | 0.006013352 | 1736/22948 | 2 |
| GO:1990173 | BP | GO:1990173 | protein localization to nucleoplasm | 2/83 | 12/18493 | 0.001275739 | 0.008161998 | 0.006013352 | 1736/22948 | 2 |
| GO:0051261 | BP | GO:0051261 | protein depolymerization | 4/83 | 105/18493 | 0.001278213 | 0.008161998 | 0.006013352 | 11004/81930/146909/3925 | 4 |
| GO:0051972 | BP | GO:0051972 | regulation of telomerase activity | 3/83 | 48/18493 | 0.001303459 | 0.008276965 | 0.006098054 | 1736/80119/9212 | 3 |
| GO:0031109 | BP | GO:0031109 | microtubule polymerization or depolymerization | 4/83 | 106/18493 | 0.001323847 | 0.008359981 | 0.006159216 | 11004/81930/146909/3925 | 4 |
| GO:0051098 | BP | GO:0051098 | regulation of binding | 7/83 | 369/18493 | 0.001345863 | 0.008452318 | 0.006227246 | 3148/3925/23560/113130/9212/1869/81620 | 7 |
| GO:0000731 | BP | GO:0000731 | DNA synthesis involved in DNA repair | 3/83 | 50/18493 | 0.0014676 | 0.009166482 | 0.006753406 | 5984/5111/51514 | 3 |
| GO:0006301 | BP | GO:0006301 | postreplication repair | 3/83 | 51/18493 | 0.001554307 | 0.00950039 | 0.006999412 | 5984/5111/51514 | 3 |
| GO:0032206 | BP | GO:0032206 | positive regulation of telomere maintenance | 3/83 | 51/18493 | 0.001554307 | 0.00950039 | 0.006999412 | 1736/9212/22948 | 3 |
| GO:0036297 | BP | GO:0036297 | interstrand cross-link repair | 3/83 | 51/18493 | 0.001554307 | 0.00950039 | 0.006999412 | 146956/2175/29089 | 3 |
| GO:0045840 | BP | GO:0045840 | positive regulation of mitotic nuclear division | 3/83 | 51/18493 | 0.001554307 | 0.00950039 | 0.006999412 | 113130/81620/11065 | 3 |
| GO:0021987 | BP | GO:0021987 | cerebral cortex development | 4/83 | 111/18493 | 0.00156922 | 0.009540523 | 0.00702898 | 9928/284403/7804/10460 | 4 |
| GO:0051656 | BP | GO:0051656 | establishment of organelle localization | 8/83 | 491/18493 | 0.001610325 | 0.009738634 | 0.007174938 | 9493/11004/3833/1062/9928/81930/113130/81620 | 8 |
| GO:1904814 | BP | GO:1904814 | regulation of protein localization to chromosome telomeric region | 2/83 | 14/18493 | 0.00174875 | 0.010520113 | 0.007750693 | 1736/22948 | 2 |
| GO:1904874 | BP | GO:1904874 | positive regulation of telomerase RNA localization to Cajal body | 2/83 | 15/18493 | 0.002011919 | 0.011933338 | 0.008791887 | 1736/22948 | 2 |
| GO:0007098 | BP | GO:0007098 | centrosome cycle | 4/83 | 119/18493 | 0.002025037 | 0.011933338 | 0.008791887 | 3832/284403/10733/6491 | 4 |
| GO:0006977 | BP | GO:0006977 | DNA damage response signal transduction by p53 class mediator resulting in cell cycle arrest | 3/83 | 56/18493 | 0.002035871 | 0.011933338 | 0.008791887 | 5111/1869/144455 | 3 |
| GO:0007062 | BP | GO:0007062 | sister chromatid cohesion | 3/83 | 56/18493 | 0.002035871 | 0.011933338 | 0.008791887 | 2237/991/113130 | 3 |
| GO:0046605 | BP | GO:0046605 | regulation of centrosome cycle | 3/83 | 56/18493 | 0.002035871 | 0.011933338 | 0.008791887 | 3832/10733/6491 | 3 |
| GO:0072431 | BP | GO:0072431 | signal transduction involved in mitotic G1 DNA damage checkpoint | 3/83 | 57/18493 | 0.002142068 | 0.012428343 | 0.009156582 | 5111/1869/144455 | 3 |
| GO:1902400 | BP | GO:1902400 | intracellular signal transduction involved in G1 DNA damage checkpoint | 3/83 | 57/18493 | 0.002142068 | 0.012428343 | 0.009156582 | 5111/1869/144455 | 3 |
| GO:0051101 | BP | GO:0051101 | regulation of DNA binding | 4/83 | 122/18493 | 0.002217314 | 0.012799947 | 0.009430361 | 3148/113130/1869/81620 | 4 |
| GO:0043486 | BP | GO:0043486 | histone exchange | 3/83 | 58/18493 | 0.002251649 | 0.012932837 | 0.009528268 | 79682/387103/55839 | 3 |
| GO:0000076 | BP | GO:0000076 | DNA replication checkpoint | 2/83 | 16/18493 | 0.00229265 | 0.012972767 | 0.009557686 | 81620/1763 | 2 |
| GO:0006206 | BP | GO:0006206 | pyrimidine nucleobase metabolic process | 2/83 | 16/18493 | 0.00229265 | 0.012972767 | 0.009557686 | 1503/7298 | 2 |
| GO:0070200 | BP | GO:0070200 | establishment of protein localization to telomere | 2/83 | 16/18493 | 0.00229265 | 0.012972767 | 0.009557686 | 1736/22948 | 2 |
| GO:0072413 | BP | GO:0072413 | signal transduction involved in mitotic cell cycle checkpoint | 3/83 | 59/18493 | 0.002364651 | 0.013184369 | 0.009713584 | 5111/1869/144455 | 3 |
| GO:1902402 | BP | GO:1902402 | signal transduction involved in mitotic DNA damage checkpoint | 3/83 | 59/18493 | 0.002364651 | 0.013184369 | 0.009713584 | 5111/1869/144455 | 3 |
| GO:1902403 | BP | GO:1902403 | signal transduction involved in mitotic DNA integrity checkpoint | 3/83 | 59/18493 | 0.002364651 | 0.013184369 | 0.009713584 | 5111/1869/144455 | 3 |
| GO:0098687 | CC | GO:0098687 | chromosomal region | 20/84 | 346/19659 | 1.82E-17 | 2.62E-15 | 1.72E-15 | 11004/1062/81930/2237/4172/332/4171/80119/5111/113130/9212/79980/81620/79682/387103/55839/147841/3070/9735/1763 | 20 |
| GO:0005819 | CC | GO:0005819 | spindle | 19/84 | 333/19659 | 1.63E-16 | 1.17E-14 | 7.72E-15 | 3832/10112/9493/24137/3833/1062/9928/56992/81930/146909/991/7443/332/284403/9212/79980/10460/79000/9735 | 19 |
| GO:0005871 | CC | GO:0005871 | kinesin complex | 11/84 | 54/19659 | 3.63E-16 | 1.74E-14 | 1.14E-14 | 3832/10112/9493/11004/24137/3833/1062/9928/56992/81930/146909 | 11 |
| GO:0005875 | CC | GO:0005875 | microtubule associated complex | 14/84 | 147/19659 | 1.84E-15 | 6.64E-14 | 4.37E-14 | 3832/10112/9493/11004/24137/3833/1062/9928/56992/81930/146909/332/7804/9212 | 14 |
| GO:0000775 | CC | GO:0000775 | chromosome centromeric region | 14/84 | 194/19659 | 8.98E-14 | 2.59E-12 | 1.70E-12 | 11004/1062/81930/332/113130/9212/79980/81620/79682/387103/55839/147841/3070/9735 | 14 |
| GO:0005874 | CC | GO:0005874 | microtubule | 18/84 | 414/19659 | 1.22E-13 | 2.94E-12 | 1.93E-12 | 3832/10112/9493/11004/24137/3833/1062/9928/56992/81930/146909/84790/3925/203068/332/9212/22948/9735 | 18 |
| GO:0000776 | CC | GO:0000776 | kinetochore | 12/84 | 133/19659 | 4.15E-13 | 8.53E-12 | 5.61E-12 | 11004/1062/81930/332/9212/79980/81620/79682/387103/55839/147841/9735 | 12 |
| GO:0000779 | CC | GO:0000779 | condensed chromosome centromeric region | 11/84 | 117/19659 | 2.65E-12 | 4.77E-11 | 3.14E-11 | 11004/1062/332/9212/79980/81620/79682/387103/55839/147841/9735 | 11 |
| GO:0000793 | CC | GO:0000793 | condensed chromosome | 13/84 | 221/19659 | 9.96E-12 | 1.59E-10 | 1.05E-10 | 11004/1062/3148/332/113130/9212/79980/81620/79682/387103/55839/147841/9735 | 13 |
| GO:0000777 | CC | GO:0000777 | condensed chromosome kinetochore | 10/84 | 104/19659 | 2.20E-11 | 3.17E-10 | 2.09E-10 | 11004/1062/332/79980/81620/79682/387103/55839/147841/9735 | 10 |
| GO:0005876 | CC | GO:0005876 | spindle microtubule | 8/84 | 54/19659 | 7.01E-11 | 9.17E-10 | 6.03E-10 | 3832/24137/1062/81930/146909/332/9212/9735 | 8 |
| GO:0072686 | CC | GO:0072686 | mitotic spindle | 8/84 | 101/19659 | 1.16E-08 | 1.39E-07 | 9.14E-08 | 3832/9493/3833/1062/81930/146909/9212/10460 | 8 |
| GO:1990023 | CC | GO:1990023 | mitotic spindle midzone | 4/84 | 13/19659 | 2.15E-07 | 2.39E-06 | 1.57E-06 | 1062/81930/146909/9212 | 4 |
| GO:0000922 | CC | GO:0000922 | spindle pole | 8/84 | 154/19659 | 3.11E-07 | 3.06E-06 | 2.01E-06 | 3832/991/284403/9212/79980/10460/79000/9735 | 8 |
| GO:0051233 | CC | GO:0051233 | spindle midzone | 5/84 | 34/19659 | 3.19E-07 | 3.06E-06 | 2.01E-06 | 1062/9928/81930/146909/9212 | 5 |
| GO:0005657 | CC | GO:0005657 | replication fork | 6/84 | 68/19659 | 4.50E-07 | 4.05E-06 | 2.66E-06 | 5984/4172/80119/5111/84296/29980 | 6 |
| GO:0030496 | CC | GO:0030496 | midbody | 8/84 | 172/19659 | 7.22E-07 | 6.12E-06 | 4.03E-06 | 10112/9493/24137/1062/9928/332/9212/11113 | 8 |
| GO:0000784 | CC | GO:0000784 | nuclear chromosome telomeric region | 6/84 | 124/19659 | 1.52E-05 | 0.000121327 | 7.98E-05 | 2237/4172/4171/80119/5111/1763 | 6 |
| GO:0000781 | CC | GO:0000781 | chromosome telomeric region | 6/84 | 158/19659 | 5.94E-05 | 0.000450185 | 0.000296174 | 2237/4172/4171/80119/5111/1763 | 6 |
| GO:0000790 | CC | GO:0000790 | nuclear chromatin | 8/84 | 369/19659 | 0.000179811 | 0.00129464 | 0.000851737 | 3148/8208/146956/3015/86/55723/113130/1869 | 8 |
| GO:0030894 | CC | GO:0030894 | replisome | 3/84 | 29/19659 | 0.000253771 | 0.001740145 | 0.001144833 | 4172/5111/29980 | 3 |
| GO:0031461 | CC | GO:0031461 | cullin-RING ubiquitin ligase complex | 5/84 | 153/19659 | 0.000503509 | 0.003295692 | 0.002168218 | 1163/991/1164/11065/51514 | 5 |
| GO:0000307 | CC | GO:0000307 | cyclin-dependent protein kinase holoenzyme complex | 3/84 | 41/19659 | 0.000713485 | 0.004467037 | 0.00293884 | 1163/898/1164 | 3 |
| GO:0043596 | CC | GO:0043596 | nuclear replication fork | 3/84 | 42/19659 | 0.000766009 | 0.004596055 | 0.00302372 | 4172/5111/84296 | 3 |
| GO:0042555 | CC | GO:0042555 | MCM complex | 2/84 | 11/19659 | 0.000967715 | 0.005574041 | 0.003667132 | 4172/4171 | 2 |
| GO:0032154 | CC | GO:0032154 | cleavage furrow | 3/84 | 54/19659 | 0.001595159 | 0.008834727 | 0.00581232 | 10112/10733/11113 | 3 |
| GO:0032993 | CC | GO:0032993 | protein-DNA complex | 5/84 | 201/19659 | 0.001706882 | 0.009103369 | 0.005989058 | 3015/4172/5111/84296/29980 | 5 |
| GO:0045171 | CC | GO:0045171 | intercellular bridge | 3/84 | 57/19659 | 0.001864487 | 0.009588792 | 0.006308416 | 10112/9493/24137 | 3 |
| GO:0032155 | CC | GO:0032155 | cell division site part | 3/84 | 63/19659 | 0.002484323 | 0.012335947 | 0.008115755 | 10112/10733/11113 | 3 |
| GO:0005881 | CC | GO:0005881 | cytoplasmic microtubule | 3/84 | 66/19659 | 0.002836563 | 0.013615503 | 0.008957568 | 11004/81930/146909 | 3 |
| GO:0032153 | CC | GO:0032153 | cell division site | 3/84 | 68/19659 | 0.003087618 | 0.014342482 | 0.009435843 | 10112/10733/11113 | 3 |
| GO:0035371 | CC | GO:0035371 | microtubule plus-end | 2/84 | 20/19659 | 0.003260601 | 0.014672702 | 0.009653094 | 11004/146909 | 2 |
| GO:0003777 | MF | GO:0003777 | microtubule motor activity | 11/83 | 84/17632 | 1.78E-13 | 3.39E-11 | 2.78E-11 | 3832/10112/9493/11004/24137/3833/1062/9928/56992/81930/146909 | 11 |
| GO:0003774 | MF | GO:0003774 | motor activity | 11/83 | 137/17632 | 4.16E-11 | 3.95E-09 | 3.24E-09 | 3832/10112/9493/11004/24137/3833/1062/9928/56992/81930/146909 | 11 |
| GO:0015631 | MF | GO:0015631 | tubulin binding | 14/83 | 325/17632 | 3.33E-10 | 2.11E-08 | 1.73E-08 | 3832/10112/9493/11004/24137/3833/1062/9928/56992/81930/146909/3925/332/22948 | 14 |
| GO:0008017 | MF | GO:0008017 | microtubule binding | 12/83 | 240/17632 | 1.27E-09 | 6.02E-08 | 4.94E-08 | 3832/10112/9493/11004/24137/3833/1062/9928/56992/81930/146909/332 | 12 |
| GO:0016887 | MF | GO:0016887 | ATPase activity | 14/83 | 445/17632 | 1.87E-08 | 7.12E-07 | 5.84E-07 | 3832/10112/9493/11004/24137/3833/1062/9928/56992/81930/146909/5984/80119/1763 | 14 |
| GO:0140097 | MF | GO:0140097 | catalytic activity acting on DNA | 8/83 | 185/17632 | 2.54E-06 | 8.06E-05 | 6.61E-05 | 2237/146956/1789/1736/80119/5111/84296/1763 | 8 |
| GO:0008574 | MF | GO:0008574 | ATP-dependent microtubule motor activity plus-end-directed | 4/83 | 26/17632 | 6.31E-06 | 0.000171169 | 0.000140349 | 3832/9928/81930/146909 | 4 |
| GO:0003684 | MF | GO:0003684 | damaged DNA binding | 5/83 | 69/17632 | 1.82E-05 | 0.000431234 | 0.000353588 | 2237/3148/1408/5111/79000 | 5 |
| GO:0043142 | MF | GO:0043142 | single-stranded DNA-dependent ATPase activity | 3/83 | 14/17632 | 3.53E-05 | 0.000744537 | 0.000610479 | 5984/80119/1763 | 3 |
| GO:1990939 | MF | GO:1990939 | ATP-dependent microtubule motor activity | 4/83 | 44/17632 | 5.37E-05 | 0.001020092 | 0.000836419 | 3832/9928/81930/146909 | 4 |
| GO:0004386 | MF | GO:0004386 | helicase activity | 6/83 | 151/17632 | 7.83E-05 | 0.001352912 | 0.001109313 | 4172/4171/80119/84296/3070/1763 | 6 |
| GO:0042393 | MF | GO:0042393 | histone binding | 6/83 | 195/17632 | 0.000315452 | 0.004994661 | 0.004095346 | 1163/8208/7443/1164/55723/4171 | 6 |

**Additional file 7-2: KEGG enrichment results of the 6 KIFs selected by LASSO regression.**

|  | ID | Description | GeneRatio | BgRatio | pvalue | p.adjust | qvalue | geneID | Count |
| --- | --- | --- | --- | --- | --- | --- | --- | --- | --- |
| hsa04110 | hsa04110 | Cell cycle | 30/87 | 124/7837 | 2.20E-33 | 1.61E-31 | 1.16E-31 | 9088/995/699/983/5347/9133/9232/891/991/8318/9700/890/9134/1869/4085/990/1870/5111/4171/8317/993/4173/10926/1111/898/4172/4175/1017/994/6502 | 30 |
| hsa03030 | hsa03030 | DNA replication | 11/87 | 36/7837 | 7.90E-14 | 2.88E-12 | 2.08E-12 | 5427/2237/5111/4171/1763/4173/5984/4172/4175/5558/5983 | 11 |
| hsa04114 | hsa04114 | Oocyte meiosis | 16/87 | 125/7837 | 3.03E-13 | 7.36E-12 | 5.31E-12 | 9088/995/699/983/5347/9133/9232/891/991/6790/9700/9134/4085/898/1017/26271 | 16 |
| hsa04914 | hsa04914 | Progesterone-mediated oocyte maturation | 13/87 | 99/7837 | 4.42E-11 | 8.07E-10 | 5.82E-10 | 9088/995/699/983/5347/9133/891/6790/890/4085/993/1017/994 | 13 |
| hsa03460 | hsa03460 | Fanconi anemia pathway | 9/87 | 54/7837 | 5.94E-09 | 8.68E-08 | 6.26E-08 | 29089/5888/55215/146956/2177/2175/641/83990/2187 | 9 |
| hsa04218 | hsa04218 | Cellular senescence | 13/87 | 160/7837 | 1.82E-08 | 2.22E-07 | 1.60E-07 | 983/9133/891/890/4605/9134/1869/1870/993/1111/898/1017/286826 | 13 |
| hsa03430 | hsa03430 | Mismatch repair | 6/87 | 23/7837 | 1.36E-07 | 1.42E-06 | 1.03E-06 | 9156/5111/5984/5983/2956/4436 | 6 |
| hsa05166 | hsa05166 | Human T-cell leukemia virus 1 infection | 13/87 | 219/7837 | 7.30E-07 | 6.66E-06 | 4.81E-06 | 9133/9232/991/9700/890/9134/1869/4085/1870/5901/1111/898/1017 | 13 |
| hsa04115 | hsa04115 | p53 signaling pathway | 8/87 | 72/7837 | 1.12E-06 | 9.06E-06 | 6.53E-06 | 983/6241/9133/891/9134/1111/898/1017 | 8 |
| hsa03440 | hsa03440 | Homologous recombination | 6/87 | 41/7837 | 5.18E-06 | 3.78E-05 | 2.73E-05 | 5888/146956/641/25788/7516/83990 | 6 |
| hsa05222 | hsa05222 | Small cell lung cancer | 8/87 | 93/7837 | 7.87E-06 | 5.22E-05 | 3.77E-05 | 1164/9134/1869/1870/898/1017/1163/6502 | 8 |
| hsa05206 | hsa05206 | MicroRNAs in cancer | 12/87 | 299/7837 | 0.000103938 | 0.000632287 | 0.000455867 | 995/113130/2146/9134/1869/1870/993/1786/3925/898/1789/994 | 12 |
